# Diclofenac Immune-Mediated Hepatitis: Identification of Innate and Adaptive Immune Responses at Clinically Relevant Doses

**DOI:** 10.3390/ijms26125899

**Published:** 2025-06-19

**Authors:** Jürgen Borlak, Reinhard Spanel

**Affiliations:** Hannover Medical School, Centre for Pharmacology and Toxicology, Carl-Neuberg-Str. 1, 30625 Hannover, Germany; spanel.r@gmx.de

**Keywords:** diclofenac, drug-induced liver injury, hepatitis, liver pathology, genomics, immuno-histochemistry

## Abstract

Diclofenac is an effective medication for pain and inflammation. However, its use has been linked to hepatitis. To gain insight into diclofenac’s ability to cause hepatitis, we investigated the regulation of major effectors of the immune system following daily treatment of minipigs at 3 and 15 mg/kg for 28 days. Histopathology evidenced lobular inflammation, and through a combination of immunogenomics and immunopathology, we detected marked innate and adaptive immune responses. We identified 109 significantly regulated genes linked to neutrophil, monocyte, Kupffer cell, and lymphocyte responses and 32 code for cytokine- and interferon-γ-signaling. In support of wound repair, immunopathology evidenced manifest upregulation of macrophage migration inhibitory factor and CD74. Furthermore, the strong expression of IgG and IgM underscored humoral immune responses. Diclofenac caused an activation of the complement system, especially the C1 inhibitor of the classical pathway and C3 with critical functions in liver regeneration. The marked expression of complement factor B and H of the alternate pathway modulated B-cell responses. Likely, the upregulation of factor H protected hepatocytes from injury by limiting complement-mediated damage of inflamed cells. Additionally, diclofenac treatment elicited marked hepatic expression of lysozyme and KLF6. The latter earmarks M1-polarized Kupffer cells. We observed an extraordinary induction of calprotectin/S100A9 and of the monocyte/macrophage CD163 scavenger receptor, and therefore, we detected innate immune sensing of damaged cells. Lastly, we noted an unprecedented induction of the acute phase reactant SAA1 and DEC-205, which recognize apoptotic and necrotic cells. Together, our results offer mechanistic insights into immune-mediated liver injury patterns following diclofenac treatment.

## 1. Introduction

Diclofenac, a nonsteroidal anti-inflammatory drug (NSAID), has been a cost-effective treatment for pain and inflammation for over four decades. Its pharmacological action primarily involves inhibition of cyclooxygenase-1 and -2, which leads to disrupted arachidonic acid metabolism and decreased prostaglandin synthesis. Additionally, diclofenac inhibits leukotriene synthesis and suppresses thromboxane-prostanoid receptor signaling [1].

Despite its effectiveness, pharmacovigilance data reveal a range of adverse drug reactions (ADRs), impacting the liver, kidneys, skin, hematological system, and nervous system. Notably, a meta-analysis of NSAID-related ADRs found that diclofenac poses vascular risks comparable to those associated with COX-2 inhibitors [2]. As a result, the European Medicines Agency’s Pharmacovigilance Risk Assessment Committee (PRAC) recommended similar precautions for diclofenac as those for COX-2 inhibitors.

The potential for diclofenac to induce liver injury (DILI) arises from several mechanisms. It is metabolized into reactive metabolites, such as quinone imines and arene oxides, which can lead to oxidative stress and significant depletion of intracellular glutathione. Additionally, diclofenac forms harmful acyl- and isoglucuronides and elicits mitochondrial toxicity [3]. These reactive metabolites can form covalent drug–protein adducts, which are recognized as neoantigens by the immune system, triggering immune responses. Although diclofenac acts as an anti-inflammatory agent, there is substantial evidence that it can provoke an inflammatory injury pattern [4,5,6,7,8]. Notably, in a prospective clinical trial involving over 17,000 arthritis patients, diclofenac frequently caused elevations in aminotransferases [9]. Furthermore, it ranked as the second most common DILI-causing agent in the general population of Iceland [10].

To explore the molecular mechanisms underlying diclofenac-induced hepatitis, we conducted a transcriptomic and histopathology study using a minipig model that is widely accepted for translational immune safety research [11]. We examined the regulation of major immune effectors in the liver of minipigs following daily treatment for 28 days. Utilizing a combination of immunogenomics and immunopathology approaches, we found compelling evidence that diclofenac elicits significant innate and adaptive immune responses at a clinically relevant dose. Our findings suggest an immune-mediated injury pattern, prompting us to characterize the specific immune cells involved.

## 2. Results

Hepatic gene expression profiling of the low- and high-dose treatments identified 153 and 488 significantly changed genes, respectively, with 70 regulated genes in common [12]. We mapped 93% of these regulated genes to the human genome and categorized the differentially expressed genes (DEGs) based on the Gene Ontology Consortium, KEGG, and BioCarta repositories. Here, we focused on the regulation of genes involved in immune and inflammatory responses, cytokine and interferon-gamma signaling, and cell death; data on significantly regulated genes are compiled in Table 1, Table 2 and Table 3.

### 2.1. Immunogenomics

Of the 488 differentially expressed genes (DEGs) identified in response to high-dose diclofenac treatment in minipigs, 109 (approximately 22%) were associated with immune and inflammatory responses, indicating that nearly one-fourth of the transcriptomic changes are linked to immunological pathways.

**Figure 1:**
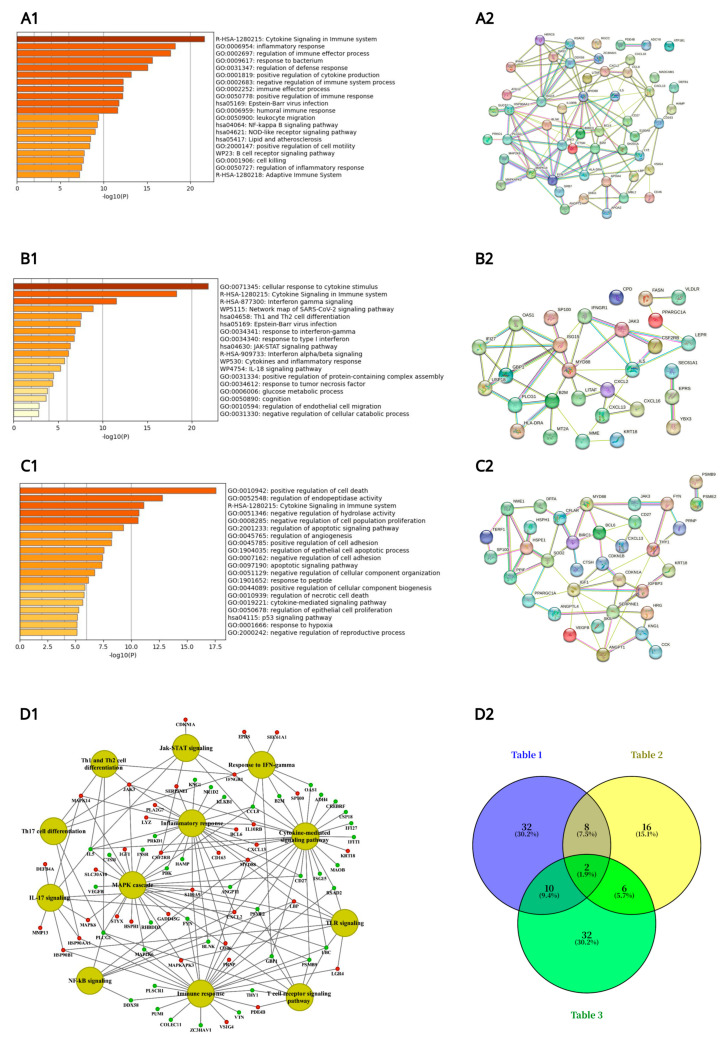
Metascape ontology analysis of immune and inflammatory gene regulations following daily diclofenac treatment of minipigs for 28 days. Depicted are Metascape pathway enrichment analysis and String protein–protein interaction (PPI) networks. **Panel** (**A1**): Pathway enrichment analysis of immune and inflammatory and leukocyte migration responsive genes listed in Table 1. **Panel** (**A2**) depicts the corresponding PPI network of DEGs listed in Table 1. **Panel** (**B1**): Pathway enrichment analysis of glucocorticoid and cytokine-mediated signaling pathway and interferon-γ-signaling responsive genes listed in Table 2. **Panel** (**B2**) depicts the corresponding PPI network of DEGs listed in Table 2. **Panel** (**C1**): Pathway enrichment analysis of cell death signaling responsive genes listed in Table 3. **Panel** (**C2**) depicts the corresponding PPI network of DEGs listed in Table 3. **Panel** (**D1**): A network of genes involved in immune and inflammatory responses. The enriched biological processes and pathways of high-dose-treated animals were computed with the Cytoscape ClueGO version 3.9 and the GeneXplain software (https://genexplain.com, accessed on 1 May 2025) and visualized using the Cytoscape software version 3.9. **Panel** (**D2**): Venn diagram to highlight common and specific immune and inflammatory response genes listed in Table 1, Table 2 and Table 3.

To explore functional associations, genes related to immune activation, inflammation, and leukocyte migration (Table 1) were analyzed using Metascape for pathway enrichment and STRING for protein–protein interaction (PPI) network construction, as illustrated in Figure 1(A1,A2). Similarly, DEGs involved in glucocorticoid signaling, cytokine-mediated signaling, and interferon-γ pathways (Table 2) underwent pathway enrichment and PPI analysis, with results shown in panels B1 and B2. Genes related to cell death and apoptosis signaling (Table 3) were evaluated using the same approach, with corresponding networks presented in panels C1 and C2.

In addition, we constructed a comprehensive gene network that focused on immune and inflammatory response-related DEGs specifically for high-dose diclofenac-treated animals using the ClueGO and GeneXplain platforms and visualized the network in Cytoscape (Figure 1(D1), see also Supplementary Figure S1 for the gene ontology and pathway mapping network of hepatic DEGs in response to low dose diclofenac treatment). Furthermore, to identify overlapping and distinct gene signatures among the three major immune responses, a Venn diagram was generated (Figure 1(D2)), providing an integrative overview of shared and pathway-specific transcriptional responses.

Specifically, the Metascape enrichment analysis highlighted terms related to inflammatory responses, positive regulation of cytokine production, cytokine and interferon γ signaling, as well as positive regulation of cell death (Figure 1), and as illustrated in Figure 1(A2), most of the significantly regulated immune and inflammatory response genes interact with one another. Similar results were obtained for the cytokine and cell death signaling networks (Figure 1(B2,C2)).

Furthermore, we created a Venn diagram (Figure 1(D2)), which revealed genes specifically linked to immune/inflammatory responses and leukocyte migration (Table 1), response to glucocorticoid stimulus, cytokine-mediated signaling pathways, and interferon γ signaling (Table 2), as well as cell death (Table 3). In total, there were 32 (19 upregulated and 13 downregulated), 16 (7 upregulated and 9 downregulated), and 32 genes (13 upregulated and 19 downregulated) specifically associated with these terms. Additionally, two genes coding for JAK3 and MYD88 were commonly regulated, as shown in the Venn diagram (Figure 1(D2)).

Among the highly regulated genes, we wish to emphasize the more than eightfold induced defensin ß1 expression (Table 1). Neutrophils are a major source of this cytotoxic peptide, and diclofenac treatment in minipigs led to a significant upregulation of neutrophils, as evidenced by blood smears and histopathological analysis using the CAE stain [12]. Diclofenac causes a mixed cholestatic liver injury pattern [13,14], and hepatocytes express defensin ß1 as well. In fact, in cholestatic liver disease, elevated bilirubin and bile acids stimulate defensin ß1 synthesis through hepatic FXR and CAR receptor signaling [15,16].

Another example relates to an induced expression of S100A9 (Table 1). This inflammatory response gene is primarily expressed in neutrophils and monocytes [17] and plays a role in stimulating leukocyte recruitment and cytokine secretion, contributing to local inflammation. Additionally, we observed a sixfold increased lysozyme expression in response to diclofenac treatment. Lysozyme is crucial for the killing of bacteria by hydrolyzing the peptidoglycan polymer in bacterial cell walls and serves as a major defense protein in the innate immune system [18]. Recent research suggested an additional immunomodulatory function for lysozyme, with serum levels elevated in patients with primary biliary cirrhosis and chronic hepatitis. Note that the protein is secreted by portal inflammatory infiltrates [19]. Following diclofenac treatment of minipigs, we observed marked lysozyme expression in damaged hepatocytes, Kupffer cells, and infiltrating monocytes (see below, immunopathology).

Furthermore, we identified 32 genes associated with cytokine and interferon γ signaling pathways, many of which are responsive to glucocorticoids (Table 2). For example, diclofenac treatment resulted in nearly a threefold induction of p21, a cyclin-dependent kinase inhibitor 1A, whose expression is stimulated by glucocorticoids [20]. Importantly, in a recent study, we demonstrated increased hepatic glucocorticoid receptor (GR) activity by immunohistochemistry (IHC), and the genomic data strongly suggest GR-dependent gene regulation [12].

Lastly, Table 3 compiles 35 significantly regulated genes associated with cell death and the regulation of cell proliferation. For instance, diclofenac treatment led to marked repression of the regulator of the cell cycle (Rgcc, Table 3). Studies in knockout mice have shown that Rgcc deficiency stimulates the proliferation of CD4+ and CD8+ T cells and induces the expression of interleukins. Consequently, Rgcc is identified as a novel regulator of T-cell activity, and its repression exacerbates the inflammatory response induced by diclofenac treatment.

### 2.2. Immunopathology

As discussed in the seminal review by Zhou and colleagues, hepatocytes play a crucial role in innate immunity [21]. To identify factors involved in diclofenac-induced hepatitis, we evaluated the regulation of various components of the innate and adaptive immune responses. Given its importance as a mediator of the innate immune response, we investigated the expression of the pro-inflammatory cytokine macrophage migration inhibitory factor (MIF) by IHC [22]. This cytokine not only blocks the protective effects of glucocorticoids [23] but also contributes to immune-mediated injury [24]. In fact, liver cells are the primary source of MIF, and their production is markedly increased during both acute and chronic injury [24]. Furthermore, MIF plays a role in chemotaxis, stimulates macrophage activity, and enhances the expression of inflammatory cytokines [22,24].

**Figure 2:**
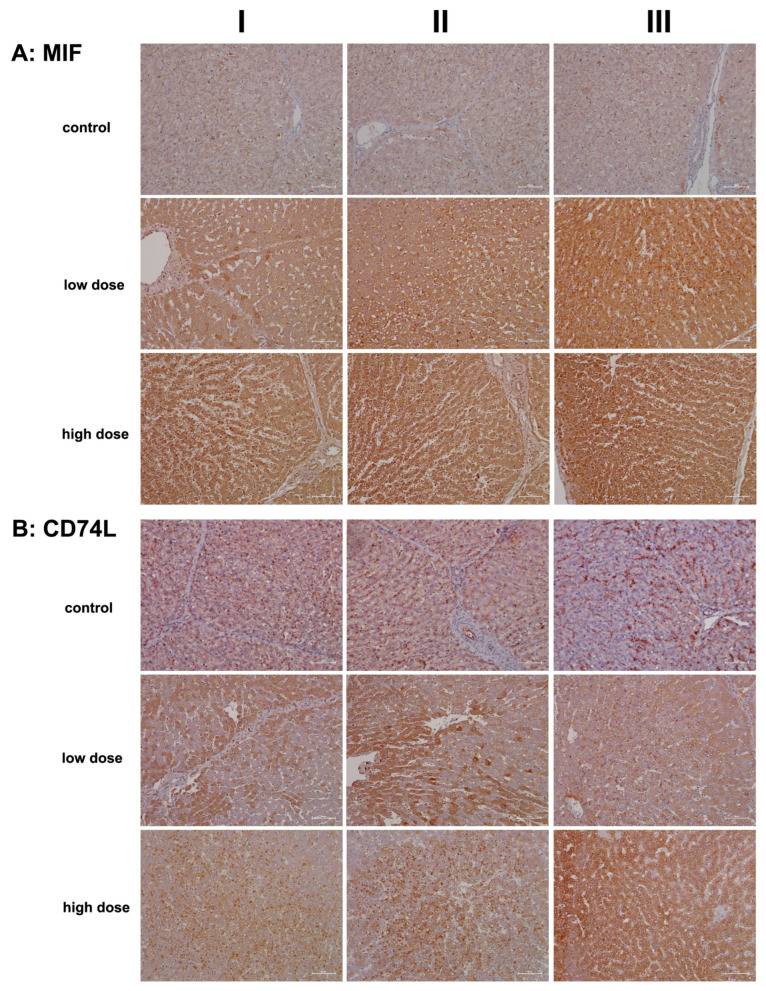
Immunohistochemistry of macrophage migration inhibitory factor (MIF) and its high-affinity receptor CD74 in liver sections of control and diclofenac-treated animals after daily dosing for 28 days. MIF is an important mediator of the innate immune response and blocks the immunosuppressive effects of glucocorticoids. MIF is a major contributor to immune-mediated injury of the liver and signals through CD74. Depicted in columns I–III are representative images of individual animals **Panel** (**A**): **MIF immunostaining: Control:** Liver sections of individual controls with minimal to slight MIF expression of resident macrophages. **Low dose:** Liver sections of low-dose-treated animals. Shown is the marked cytosolic expression of MIF1 in inflamed hepatocytes and infiltrating Kupffer cells. **High dose:** Liver sections of high-dose-treated animals with marked MIF1 expression. **Panel** (**B**)**: CD74L: Control:** Liver sections of individual controls with slight to moderate CD74 expression of lymphocytes, macrophages, and dendritic cells. The dust-like appearance of CD74-positive cells characterizes hepatic stellate cells (HSC) in the space of Disse. Typically, HSC cannot be seen by light microscopy. **Low dose:** Liver sections of low-dose-treated animals with highly induced CD74 expression of regenerating liver cells. **High dose:** Liver sections of high-dose-treated animals. Shown are inflamed liver lobules with marked CD74 expression of Kupffer cells and lymphocytes (columns I–II). Furthermore, the image shown in column III highlights the hepatocytic expression of this protein. The scale bar represents 50 µm.

Figure 2A presents representative liver sections from control animals (columns I–III). Cytosolic expression of MIF is generally faint, though a few hepatocytes (column III) exhibit more prominent staining. The sinusoids display slight to moderate MIF positivity, and Kupffer cells are also positive, albeit with variable staining intensity.

In contrast, diclofenac treatment at both the low and high doses resulted in marked cytosolic expression of MIF. Some hepatocytes in the hypertrophied zone 1 exhibited enhanced MIF expression (low dose, column I). Additionally, activated macrophages displayed strong expression of the protein (low dose, column II; high dose, column II). The liver sections also revealed sinusoidal dilation, swollen endothelial cells, edema, and widening of the space of Disse, which are indicative of liver regeneration following daily diclofenac treatments for 28 days.

The data suggest that damaged hepatocytes recruit MIF-positive monocytes along with other leukocytes (low dose, column III; high dose, columns I–III) to areas of injury characterized by focal cell lysis. Interestingly, one report proposed a protective role for MIF in fatty liver degeneration. Given that diclofenac also induces steatosis, its interaction with its receptor, CD74, is critical, as discussed below [25].

Figure 2B shows representative liver sections from control animals (columns I–III). While hepatocytes are negative for CD74, distinct CD74-positive seed-like structures or endosomes are clearly visible. CD74 plays a multifaceted role, notably in antigen presentation and in the assembly and intracellular trafficking of the MHC class II complex [26]. Additionally, it induces endosomal fusions and plays a role in delivering endosomal cargo destined for lysosomal degradation [27].

Hepatic stellate cells (HSCs) exhibit a dust-like appearance and are stained positive for CD74. These cells are localized in the space of Disse, which lies between hepatocytes and sinusoids, and they are typically undetectable by light microscopy. Furthermore, endothelial cells (column II, artery) express CD74, albeit with varying intensities.

Low-dose diclofenac treatment (columns I–III) resulted in marked cytosolic expression of CD74, with liver sections displaying a speckled pattern of CD74-positive and -negative hepatocytes (columns I–II). In contrast, the high-dose regimen induced a significant increase in CD74-positive endosomes, reflecting enhanced endosomal trafficking of endocytosed cellular debris targeted for lysosomal degradation (columns I–II). Additionally, diclofenac treatment caused hepatic steatosis, with some lipid vacuoles exhibiting positive CD74 staining.

To investigate humoral immune responses in the liver sections of diclofenac-treated animals, we conducted staining for IgG and IgM. While immunoglobulins are primarily produced by B cells and plasma cells, there is evidence that hepatocytes can also synthesize immunoglobulins, including IgG [28] and IgM [29], as well as immunoglobulin A-containing vesicles within hepatocytes [30,31]. A recent review summarized some of the molecular events involved in immune-mediated drug-induced liver injury [32]. Importantly, serum immunoglobulins have diagnostic value; for instance, IgG levels are elevated in autoimmune hepatitis (AIH), while IgM levels are increased in primary biliary cholangitis (PBC) [33].

**Figure 3:**
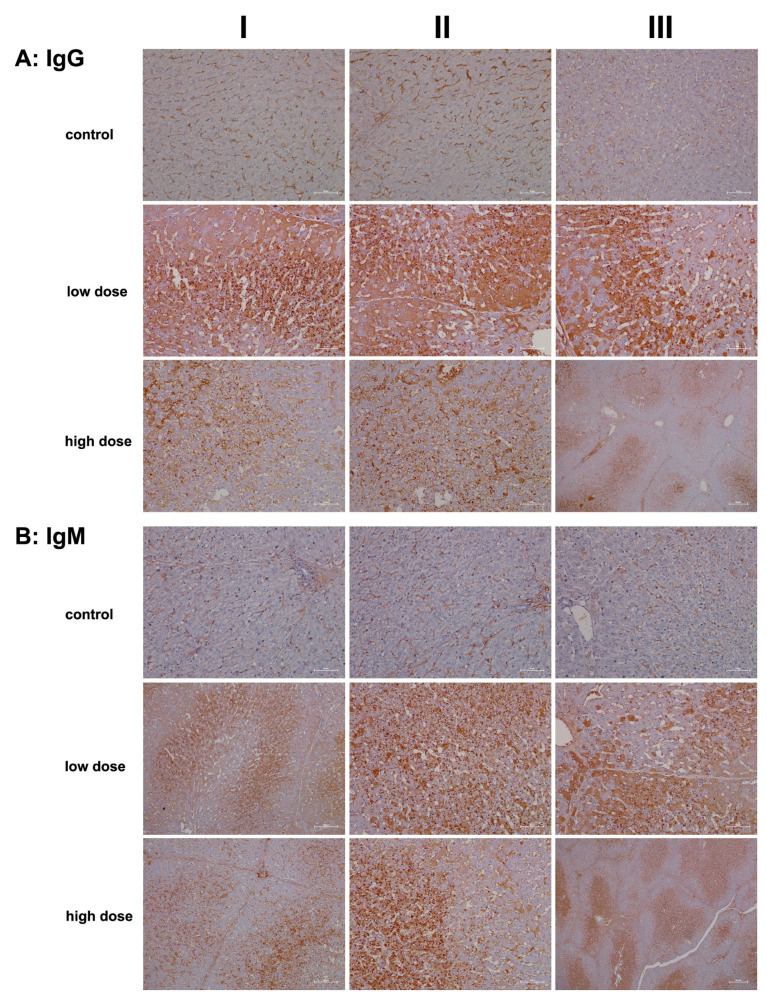
Immunohistochemistry of IgG and IgM in liver sections of control and diclofenac-treated animals after daily dosing for 28 days. IgG and IgM are part of the drug antibody response. Reactive diclofenac metabolites form protein adducts, which are sensed by immunosurveillance as neoantigens and elicit B-cell responses with the production of drug antibodies. Depicted in columns I–III are representative images of individual animals. **Panel** (**A**): **IgG immunostaining: Control:** Liver sections of individual controls. Only the sinusoids stained positive. **Low dose:** Liver sections of low-dose-treated animals with marked hepatic synthesis of IgG and an extraordinary infiltration of IgG-positive B cells (columns I–II) into zones of hepatic injury. **High dose**: Liver sections of high-dose-treated animals. Diclofenac treatment caused marked IgG-positive lymphocytic infiltrates; however, severely damaged hepatocytes synthesized less IgM. Low-dose treatment (column III) caused an apparent zonation of IgM-positive hepatocytes, along with B-cell infiltrates. **Panel** (**B**): **IgM immunostaining: Control**: Liver sections of individual controls. Only the sinusoids stained positive. **Low dose:** Liver sections of low-dose-treated animals with marked hepatic synthesis of IgM. Shown is the zonation of IgM-positive hepatocytes and IgM-positive B-cell infiltrates. **High dose:** Liver sections of high-dose-treated animals with marked hepatic synthesis of IgM. The findings are similar to the ones at the low-dose diclofenac treatment. Therefore, diclofenac treatment elicits a dose-independent bold immune response. The scale bars represent 50 µm unless otherwise indicated. Exceptions include **panel** (**A**), high dose, column III (200 µm); **panel** (**B**), low and high dose, column I (100 µm); and **panel** (**B**), high dose, column III (200 µm).

Representative liver sections from controls (Figure 3A, columns I–III) exhibit IgG positivity exclusively in the sinusoids. By comparison, low-dose diclofenac treatment resulted in marked hepatic IgG synthesis (columns I–III), with variable staining intensity among hepatocytes. The immunoreactive dot-like structures likely represent endosomes, which may be involved in an autophagy process, including the endocytosis of cellular debris. Another finding is the interlobular zonation of IgG-positive hepatocytes (column III).

Next, we investigated IgM expression. Typically, IgM responses are the first to arise following exposure to an immunogen. IgM is highly effective in opsonizing antigens, while IgG triggers complement activation and specifically Fcγ receptor responses. In liver sections of controls (Figure 3B), only the sinusoids stained positive for IgM. Remarkably, low-dose diclofenac treatment induced significant hepatic synthesis of IgM (columns I–III), with the immunoreactive endosomes (dot-like structures) indicating autophagy and endocytosis of cellular debris, alongside lipid vacuoles that also stained positively. Additionally, we observed B-cell and monocytic infiltrates in the zones of hepatic injury (panel columns I–II), indicating that diclofenac treatment led to lobular inflammation, even though the synthesis of IgM varied among hepatocytes. In fact, not all hepatocytes expressed the protein.

At the higher dose (columns I–III), severely damaged and vacuolar-degenerated hepatocytes exhibited reduced synthesis of IgM or failed to produce it altogether. Nonetheless, the positively stained endosomes (low dose; column II and high dose, columns I–II) highlight the endocytosis of cellular debris and its subsequent lysosomal degradation. The image shown in column III of the high dose illustrates a distinct zonation of IgM-positive hepatocytes, with B-cell infiltrates migrating toward the boundary of the liver lobule. Overall, diclofenac treatment caused robust IgG and IgM immune responses.

Given its crucial role in regulating the innate immune response and its anti-inflammatory properties, we investigated the regulation of the C1 inhibitor, i.e., a major inhibitor of the classical complement pathway [34,35,36]. We also evaluated the regulation of complement component C3, which is essential for activating the innate immune response within the complement cascade [37].

**Figure 4:**
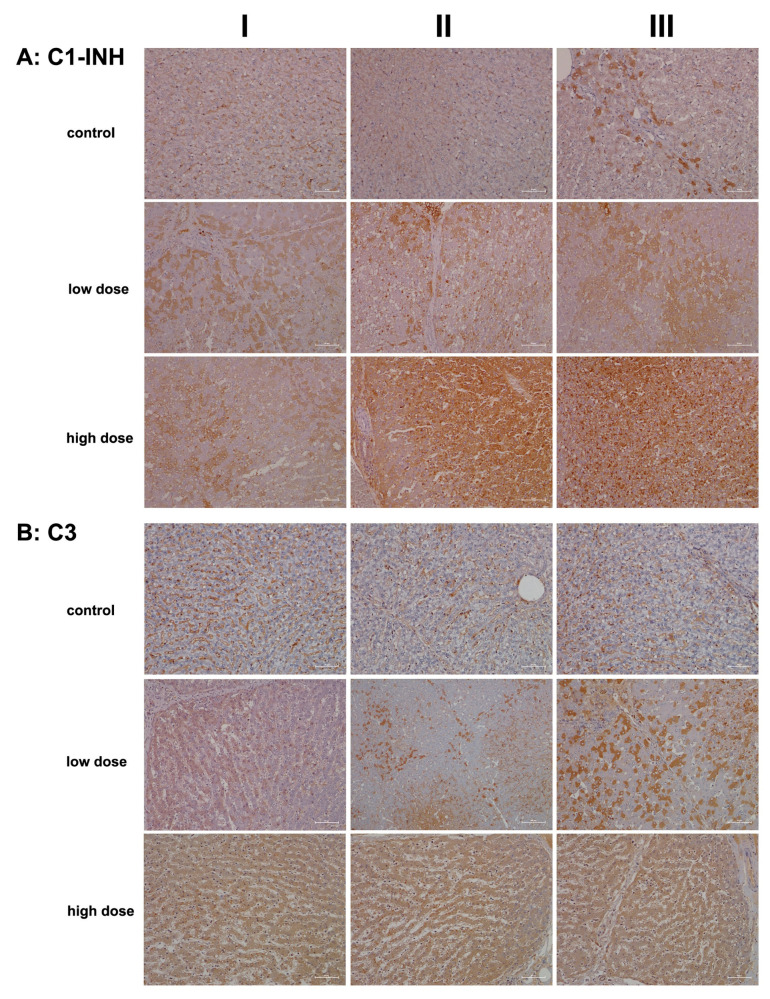
Immunohistochemistry of C1 inhibitor and complement factor C3 in liver sections of control and diclofenac-treated animals after daily dosing for 28 days. The C1 inhibitor and C3 are factors of the classical and common terminal pathway of the complement system, and their regulation demonstrates an erroneous programming of the immune response by diclofenac. Depicted in columns I–III are representative images of individual animals. **Panel** (**A**): C1 inhibitor immunostaining. Controls: Liver sections of individual controls. Only the sinusoids stained positive. However, column III illustrates a liver section from a single animal, where limited expression of the protein is observed in zone 1 hepatocytes. **Low dose:** Liver sections of low-dose-treated animals. Regenerating liver cells induce C1 inhibitor expression to block complement activation (column I). However, severely harmed hepatocytes do not express the protein (columns II–III). Kupffer cells strongly express the C1 inhibitor (column II). **High dose:** Liver sections of high-dose-treated animals. The hepatic synthesis of the C1 inhibitor is markedly and dose-dependently increased (columns II–III). Within an inflamed liver lobule, monocytic infiltrates and resident macrophages strongly express the protein (column III). **Panel** (**B**): **Complement factor C3 immunostaining:** Liver sections of individual controls. Apart from sinusoidal endothelial cells and occasionally resident macrophages, very rarely, hepatocytes are C3 positive. **Low dose:** Liver sections of low-dose-treated animals. Diclofenac treatment caused a heterogeneous (columns I–II) and mosaic-like expression pattern (column III) of complement C3. Regenerating hepatocytes express C3 more abundantly. **High dose:** Liver sections of high-dose-treated animals. Diclofenac treatment caused a dose-related increase in C3 protein synthesis. The scale bar represents 50 µm, except for **panel** (**B**), low dose, column II, where it represents 100 µm.

Figure 4A presents representative liver sections from control animals (columns I–III), demonstrating C1 inhibitor expression predominantly in sinusoidal endothelial cells, with occasional hepatocyte staining (column III). Diclofenac treatment led to a dose-dependent increase in hepatic C1 inhibitor synthesis, reflecting precise regulation of the complement system.

At the high treatment dose (columns II–III), we observed marked increases in immunoreactive vacuoles and endosomes and speculate these to be part of an autophagy process. Additionally, some Kupffer cells, particularly in areas of severely damaged and vacuolar-degenerated hepatocytes, expressed the C1 inhibitor protein (columns II–III). Bile duct epithelial cells stained positive for the C1 inhibitor protein as well (column II).

In addition to being the primary source of the C1 inhibitor protein, hepatocytes also synthesize complement component C3. This multifunctional protein has been rightly described as the “Swiss Army Knife of innate immunity and host defense”, a phrase coined by Ricklin and colleagues [37]. C3 is critical for liver regeneration [38], and endothelial cells contribute to its local production [39].

Figure 4B illustrates representative images of liver sections from controls. Here, C3 was primarily detected in the endothelial cells of the sinusoids, with rare instances of positively stained hepatocytes. Diclofenac treatment resulted in a dose-dependent increase in C3 synthesis. Columns II–III show a mosaic-like C3 expression pattern in low-dose-treated animals, with column III depicting an inflamed portal field featuring intact bile ducts and C3-positive macrophage infiltrates.

At the higher dose, most hepatocytes actively synthesized C3, particularly in zones 1 and 2, which exhibited more injury. Notwithstanding, some liver cells demonstrated a greater capacity for regeneration. The immunoreactive dot-like structures are indicative of an autophagy process. Diclofenac induces metabolic disorders, and the resulting steatosis triggers lipophagy. Interestingly, recent studies suggest that intracellular C3 may help prevent or reduce hepatic steatosis by promoting autophagy and very-low-density lipoprotein secretion [40].

Given the contrasting roles of these two proteins—an anti-inflammatory function of the C1 inhibitor and the immunomodulatory role of C3—a complex picture emerges where diclofenac both stimulates and mitigates acute inflammatory responses. Additionally, C3 is recognized as a vital driver of liver regeneration [41,42].

**Figure 5:**
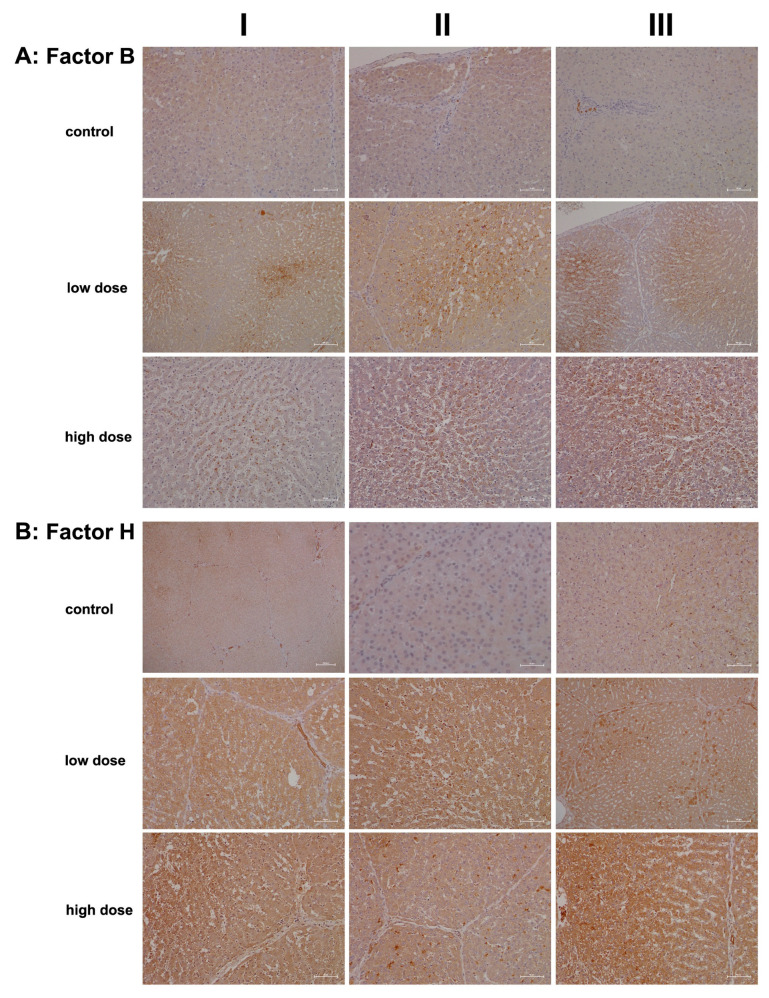
Immunohistochemistry of complement factor B and H in liver sections of control and diclofenac-treated animals after daily dosing for 28 days. Factor B and H are essential components of the alternate pathway. Depicted in columns I–III are representative images of individual animals. **Panel** (**A**): **Complement factor B immunostaining. Control:** Liver sections of individual controls. The cytosol of hepatocytes stained faintly positive. The liver section in column III depicts a group of factor B+ macrophages within a portal triad. **Low dose:** Liver sections of low-dose-treated animals. Diclofenac treatment induced factor B expression in hepatocytes and Kupffer cells. Its expression is confined to zone 2 and 3 hepatocytes and factor B-positive Kupffer cells cluster around zones of inflammation. **High dose**: Liver sections of high-dose-treated animals. Diclofenac treatment caused marked induction of factor B expression in monocytes and resident macrophages. Unlike the low-dose treatment, where hepatocytes also express the protein, factor B synthesis in the high-dose regimen is primarily confined to immune cells. **Panel** (**B**): **Complement factor H immunostaining. Control:** Liver sections of controls. Except for biliary epithelium, neither hepatocytes nor resident immune cells express factor H. **Low dose:** Liver sections of low-dose-treated animals. Diclofenac treatment caused marked expression of factor H in hepatocytes, Kupffer cells, and biliary epithelium. Note the mosaic-like expression pattern among hepatocytes (column III). Given its protective function on self-cells, and to prevent an indiscriminate immune response, it appears that some hepatocytes are particularly efficient in protecting themselves from immune-mediated injury. **High dose**: Liver sections of high-dose-treated animals. The findings are similar to the ones obtained with the low-dose treatment, and the regulation of complement factor H appears to be dose-independent. The scale bar represents 50 µm, except for the following: **panel** (**A**), low dose, columns I and III (100 µm); **panel** (**B**), control, column I (200 µm) and column II (25 µm); and **panel** (**B**), low dose, column III (100 µm).

We also investigated whether diclofenac treatment activates the alternative complement pathway and therefore examined the regulation of complement factors B and H (Figure 5). Both factors play crucial roles in the control of this pathway: factor B stimulates B-cell activation and differentiation of antigen-activated B cells. It is synthesized and secreted by various immune cells, including polymorphonuclear cells (PMCs), monocytes, macrophages, dendritic cells, and T cells, as well as hepatocytes [21,43]. In contrast, factor H recognizes host cell markers and protects cells from injury by limiting complement-mediated damage in inflamed or injured tissues. It is synthesized by many cell types, including hepatocytes, endothelial cells, epithelial cells, and platelets [44].

Figure 5A shows representative images of liver sections from controls. Only a few macrophages (column III, portal field) expressed factor B, while hepatocytes and endothelial cells were negative. Conversely, treatment with diclofenac at the low dose led to an upregulation of factor B synthesis in resident macrophages and monocytes, indicating activation of the alternative complement pathway.

Unlike C1 inhibitor and C3, the regulation of factor B appeared subtler and may have resulted from hepatic steatosis induced by diclofenac [45,46]. This factor supports regeneration by blocking cellular senescence, as has been observed in murine and human pancreatic epithelial cell lines [47]. Additionally, the non-obstructive sinusoidal dilatation may stem from impaired portal perfusion due to an acute inflammatory response and is similar to findings with other drugs [48].

Particularly for the low-dose treatment, expression of factor B was confined to hepatocytes of zones 2 and 3. Furthermore, the staining pattern indicated the presence of a “complosome”, i.e., a recently discovered concept that describes complement factors with non-canonical functions in autophagy [49].

Shown in Figure 5B are liver sections of controls stained for complement factor H. This factor plays a critical role in the alternative pathway by blocking its amplification loop. The cytosol of hepatocytes stained slightly positive, while bile duct epithelial cells showed marked expression (column I). Occasionally, macrophages stained positive as well (column III). Treatment with diclofenac led to significant hepatic synthesis of factor H; however, this increase was not dose-dependent. Bile duct epithelial cells, Kupffer cells, and certain hepatocytes expressed factor H at varying intensities, while the sinusoids were mostly negative (low dose, columns I–II). Occasionally, we observed a speckled expression pattern of factor H (column III); therefore, some hepatocytes expressed factor H more abundantly than others. The liver section shown in column II illustrates focal inflammatory infiltrates within the sinusoids of a liver lobule, along with accompanying edema and fresh lytic cellular necrosis. Zonal liver regeneration is exemplified by shrunken hepatocytes in zones 2 and 3, suggesting a replacement of necrotic liver cells (see also immunostaining for factor B). Additionally, lipid vacuoles align pearl-like along the sinusoids, similar to the pattern seen for the IgM stain (Figure 3, high dose, column II). Less harmed hepatocytes tend to express factor H more abundantly (low dose, column III). Given its function in blocking the amplification loop and the formation of C3 and C5 convertases, we interpret its upregulation as supportive of liver regeneration following diclofenac treatment.

Another important member of the innate immune system is the lysozyme, which is synthesized and secreted by macrophages, neutrophils, and dendritic cells. Cytokines also stimulate its synthesis by hepatocytes [50,51,52]. In addition to its antimicrobial function, which involves hydrolysis of bacterial cell walls, there is evidence that lysozymes modulate immune responses by stimulating and limiting inflammatory reactions [18,52]. Following diclofenac treatment, we observed an up to sixfold increase in lysozyme gene expression in liver tissue extracts (Table 1), prompting our interest in investigating its expression through immunohistochemistry (IHC).

**Figure 6:**
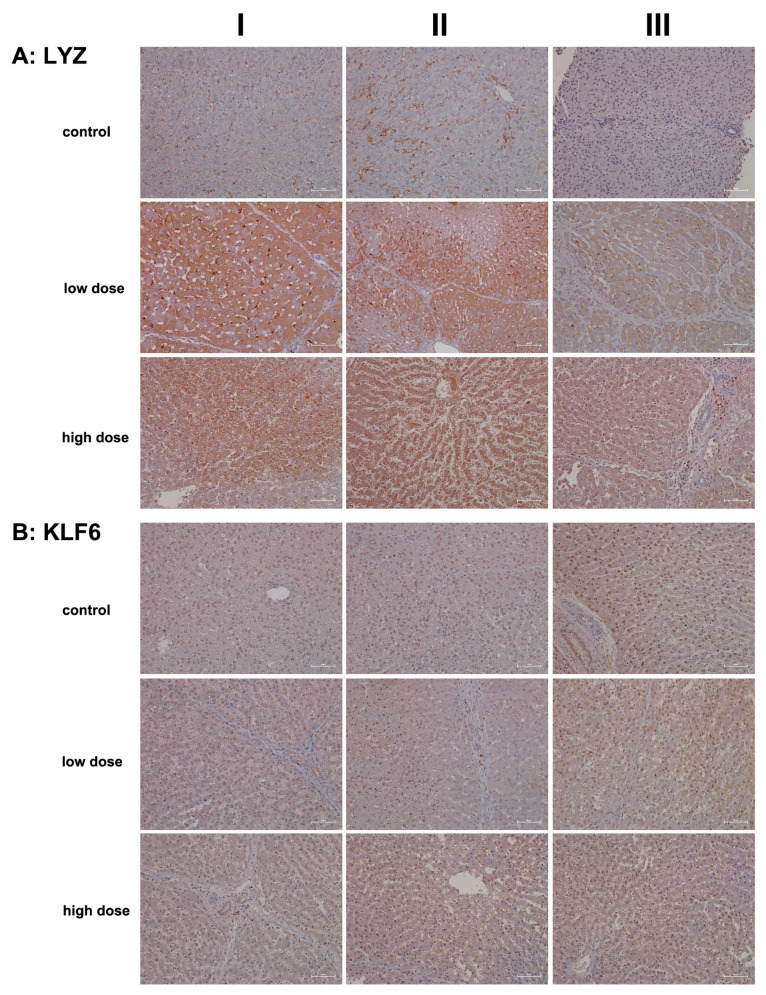
Immunohistochemistry of lysozyme and Krüppel-like factor 6 (KLF6) in liver sections of control and diclofenac-treated animals after daily dosing for 28 days. Lysozyme activation appears paradoxical in “sterile” inflammation, but recent evidence suggests that new functions result in the activation of pattern recognition receptors of host cells. Furthermore, KLF6 is strongly induced by pro-inflammatory stimuli and earmarks M1-polarized macrophages. Depicted in columns I–III are representative images of individual animals. **Panel** (**A**): **Lysozyme immunostaining. Control**: Liver sections of individual controls. The sinusoids as well as resident macrophages (columns I–II) stained positive; however, hepatocytes do not express the lysozyme protein (column III). **Low dose**: Liver sections of low-dose-treated animals. Diclofenac treatment led to an induced lysozyme expression in hepatocytes and macrophages but not all hepatocytes express the protein. **High dose**: Liver sections of high-dose-treated animals. The findings are similar to the ones obtained at the low dose, and therefore, lysozyme expression is not dose-related. Notwithstanding, especially severely harmed hepatocytes express lysozyme more abundantly to possibly direct cytotoxic immune cells to zones of injury, thereby aggravating the immune response. **Panel** (**B**): **KLF6 immunostaining**. **Control**: Liver sections of individual controls; except for a single control (column III) with slight positive KlF6 nuclear staining, none of the hepatocytes and resident macrophages expressed the protein. **Low dose:** Liver sections of low-dose-treated animals. Depicted are KLF6-positive macrophages in a portal field (columns I–II). Some of the liver cell nuclei stained slightly positive (column III). **High dose:** Liver sections of high-dose-treated animals. The findings are similar to those observed at the low dose. Diclofenac treatment caused a heterogeneous response among resident macrophages. KLF6-positive monocytes infiltrate a portal field (high dose, column I). Occasionally, Kupffer cells in the sinusoidal space of treated animals stained positive as well (columns II–III). The nuclei of hepatocytes are faintly positive. The scale bar represents 50 µm, except for **panel** (**A**), low dose, column II, where it represents 100 µm.

Figure 6A shows representative images of liver sections from two controls stained for lysozyme, while the image in column III depicts a liver biopsy from another control animal. The sinusoids and macrophages/monocytes (columns I–II) exhibited positive staining at varying intensities; however, hepatocytes do not express the lysozyme protein. In contrast, diclofenac treatment at the low dose led to significantly induced hepatic synthesis of lysozyme, though not all hepatocytes exhibited the protein to the same extent. The liver section shown in column III depicts an inflamed and degenerated liver lobule in which macrophages strongly expressed lysozyme. At the high dose and apart from induced hepatic synthesis of lysozymes (columns I–II), we observed focal, lysozyme-positive mixed inflammatory infiltrates in portal triads (column III).

To investigate the role of KLF6 as a regulator of macrophage polarization (specifically M1), we assessed its expression levels. Previous work indicated induced KLF6 expression in macrophages in a canine model of immune-mediated hepatitis [5]. KLF6 functions by suppressing anti-inflammatory gene expression through the repression of PPARγ [53]. In the present study, we observed repressed PPARγ transcriptional activity, particularly at the high dose (Table 1). Furthermore, we noted activation of interferon γ-mediated signaling, which supports both innate and adaptive immune responses following diclofenac treatment [5].

Shown in Figure 6B are liver sections from controls, and the nuclei of hepatocytes and bile duct epithelium (column III) exhibit varying intensities of KLF6 positivity. While Kupffer cells in control animals do not express the protein, the cytosol of hepatocytes stained slightly positive at both low- and high-dose treatments. Clearly, KLF6 expression was dose-independent, and columns I–II (low dose) and column I (portal fields, high dose) exemplify a few Kupffer cells with prominent KLF6 expression, thus indicating a pro-inflammatory (M1) state.

To illustrate diclofenac’s ability to induce immune-mediated hepatitis, we investigated the regulation of the S100A9 protein. This calcium-binding protein was induced >fourfold in the livers of diclofenac-treated minipigs (Table 1) and plays a crucial role in inflammatory signaling cascades, known as “alarmins”. Importantly, S100A9 is mainly expressed in cells of myeloid origin (neutrophils, monocytes, eosinophils) and facilitates the migration of myeloid cells to sites of injury [17,54,55]. Further evidence for an upregulation of chemotactic factors is listed in Table 1, with an induced expression of CXCL2 and CXCL13. Intriguingly, hepatocyte-specific S100A8 and S100A9 transgene expression in mice caused CXCL1 induction and systemic neutrophil enrichment [56], and similar to CXCL1, the chemokines CXCL2 and CXCL13 promote neutrophil and B-cell migration and are mainly produced by monocytes/macrophages [55]. Given the predominant expression of S100A8 and S100A9 in immune cells, we were astonished to observe positive staining of inflamed hepatocytes. Nonetheless, research identified S100A9 as part of alarm signals termed “damage-associated molecular patterns” (DAMPs), which can induce sterile inflammation independently of RAGE and TLR4 [56,57] and function in inflammation and immune response within the cell [58].

**Figure 7:**
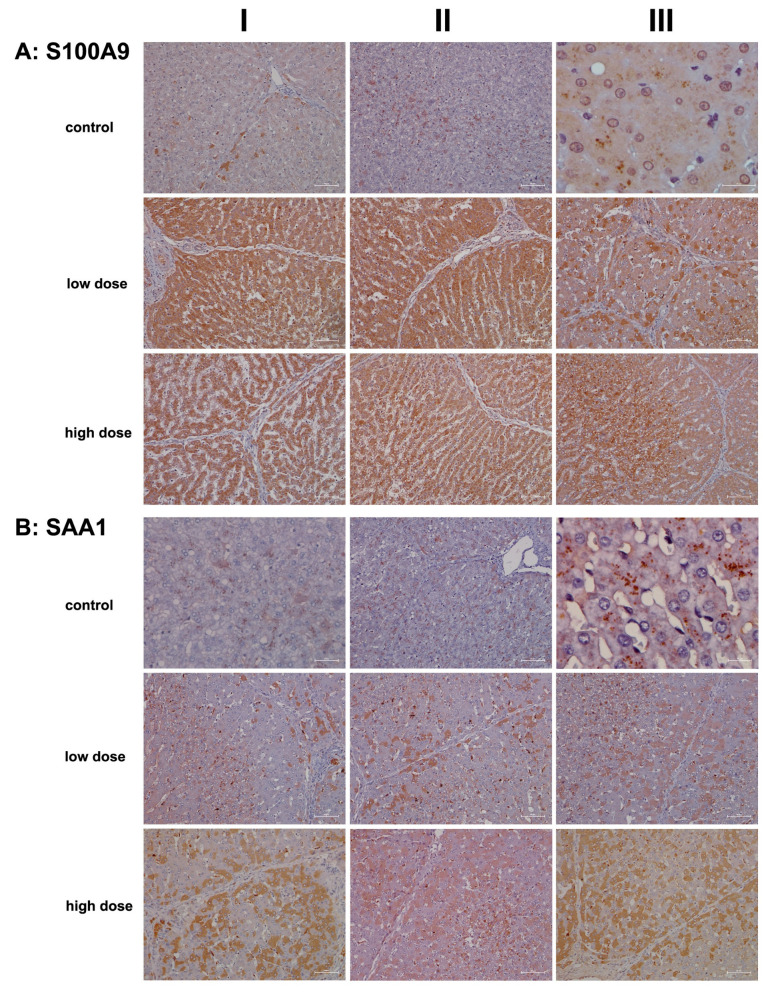
Immunohistochemistry of S100A9 and SAA1 in liver sections of control and diclofenac-treated animals after daily dosing for 28 days. The Ca2+ binding protein S100A9 and the acute phase reactant SAA1 are key players in the immune response and, in part, function in the chemotactic migration of immune cells and hepatic stellate cells to sites of injury. Depicted in columns I–III are representative images of individual animals. **Panel** (**A**): **S100A9 immunostaining**. **Control:** Liver sections of individual controls. Except for a few hepatocytes (columns I–II), none of the liver cells and resident macrophages express the S100A9 protein (AIII). We observed S100A9 positive vesicles (column II, low magnification; column III, high-power field magnification), which likely represents an endolysosomal storage compartment of this protein. **Low dose:** Liver sections of low-dose-treated animals. Diclofenac treatment caused an extraordinary expression/synthesis of the S100A9 protein in hepatocytes and resident macrophages (columns I–II). Apparently, regenerating hepatocytes are devoid or express less of the S100A9 protein (column III). **High dose**: Liver sections of high-dose-treated animals. Essentially, the findings are similar to the ones obtained at the low-dose treatment, and therefore, the expression of the S100A9 protein is not dose-related (columns I–II). The image in column III exemplifies a liver section of a high-dose-treated animal. Note the severely harmed hepatocytes (mainly in zones 2 and 3 of the hepatic lobule) with marked expression of the S100A9 protein. **Panel** (**B**): **SAA1 immunostaining. Control:** Liver sections of individual controls. A few hepatocytes with pre-apoptotic alterations express SAA1 faintly (column I). We observed storage-like SAA1-containing particles (columns II–III). Importantly, the liver is a major site for the synthesis of nascent HDL [60], and SAA binds to the lipid surface of HDL particles. **Low dose:** Liver sections of low-dose-treated animals. Some hepatocytes express SAA1 abundantly in support of leucocyte infiltration and neutrophil adhesion to zones of inflammation and to stimulate the secretion of various cytokines. Activated HSC and resident macrophages are SAA1-positive. **High dose:** Liver sections of high-dose-treated animals. Depicted are inflamed liver lobules with marked cytosolic expression of the SAA1 protein. Not all hepatocytes are SAA1-positive (columns I–III), and the liver section shown in column II illustrates the significant infiltration of an inflamed liver lobule by activated and SAA1-positive HSC and Kupffer cells. The scale bar represents 50 µm, except for **panel** (**A**), control, column III (20 µm); **panel** (**B**), control, column I (25 µm); and **panel** (**B**), control, column III (10 µm).

As shown in Figure 7A hepatocytes from controls do not express S100A9 except under stressed conditions (columns I–II). Note the S100A9-positive vesicles in liver sections of controls (columns II–III), which likely represent an endo-lysosomal storage compartment of this protein, as suggested by Chakraborty and colleagues [59]. We observed positive staining of hepatocytes following diclofenac treatment at both the low- and high-dose treatments. While its expression is not clearly dose-related, less harmed hepatocytes showed reduced S100A9 expression compared to severely harmed ones (low dose, column III). Depicted in column III is the liver section from a low-dose-treated animal, demonstrating lobular inflammation and a zonated pattern of S100A9-positive inflammatory cell infiltration.

We also examined the expression of serum amyloid A1 (SAA1), a major acute phase reactant typically upregulated in response to tissue injury and infection. Following diclofenac treatment, SAA1 is primarily synthesized in the liver and plays multiple roles, including facilitating immune cell migration, cytokine and chemokine production, and interaction with Toll-like receptors to activate NF-κB and mitogen-activated protein kinases [60,61]. Recent studies highlighted the SAA1/TLR2 axis in directing the chemotactic migration of hepatic stellate cells to injury sites [61].

Figure 7B presents representative liver sections from control animals. As expected under non-stressed conditions, hepatocytes showed minimal SAA1 expression. The image in column I features faintly SAA1-positive hepatocytes with pyknotic nuclei, while the image in column III reveals storage-like SAA1-containing particles. Importantly, the liver produces nascent HDL, and SAA binds to its lipid surface [62].

We observed a dose-dependent increase in hepatic SAA1 synthesis following diclofenac treatment. Liver sections from individual low-dose-treated animals reveal lobular inflammation, SAA1-positive macrophages throughout, and a cluster of SAA1-positive mixed immune cells within a portal field (column I). However, not all hepatocytes expressed the protein, with zone 1 hepatocytes appearing more engaged, and activated Kupffer cells expressed SAA1 abundantly.

Images shown in columns I–III present liver sections from high-dose-treated animals, highlighting the dose-dependent increase in hepatic SAA1 synthesis. The protein displayed a mosaic expression pattern, with column II showing inflamed regions enriched in SAA1-positive immune cells and comparatively weaker staining in adjacent hepatocytes.

Collectively, our findings suggest that SAA1 exacerbates inflammation. At the low dose, predominantly zone 2 and 3 hepatocytes are engaged, whereas at the high dose, hepatocytes across all zones are affected, indicating that zone 1 hepatocytes may participate in a compensatory growth response.

C-type lectin receptors (CLRs) are pattern recognition receptors (PRRs) that recognize molecules expressed by damaged cells, commonly referred to as alarmins or DAMPs. Through the sensing of these DAMPs, CLRs prompt an immune response. Specifically, lymphocyte antigen 75, also known as DEC-205 or CD205, serves as a recognition receptor for apoptotic and necrotic cells [63]. This mannose receptor belongs to the type 1 family of C-type lectins, characterized by multiple lectin domains in its extracellular region [64]. DEC-205 is predominantly expressed in monocytes, myeloid dendritic cells, B cells, NK cells of peripheral blood mononuclear cells (PBMCs), and various tissue macrophage subpopulations [65]. Research has established an essential role for DEC-205/CD205 in antigen processing and cross-presentation via MHC molecules, thereby stimulating T-cell responses [63,64].

On the other hand, CD302 is a single-domain C-type lectin receptor that functions as a fusion protein encoded by an intergenically spliced mRNA of DEC-205 and CD302 [66]. Also known as LY75-CD302 or the DEC-205-associated CLR fusion protein, CD302 is highly expressed in macrophages, granulocytes, and dendritic cells. Additionally, CD302 supports the migration of myeloid dendritic cells to injury sites [67,68]. Note that hepatocytes and liver sinusoidal endothelial cells also express CD302, particularly in response to LPS-induced inflammation [68].

We used an antibody that recognizes an epitope at the N-terminus of DEC-205, which also cross-reacts with the same epitope on the DEC-205-associated CLR fusion protein. This approach allowed us to assess the regulation of both receptors, DEC-205 and CD302, through immunohistochemistry (IHC).

**Figure 8:**
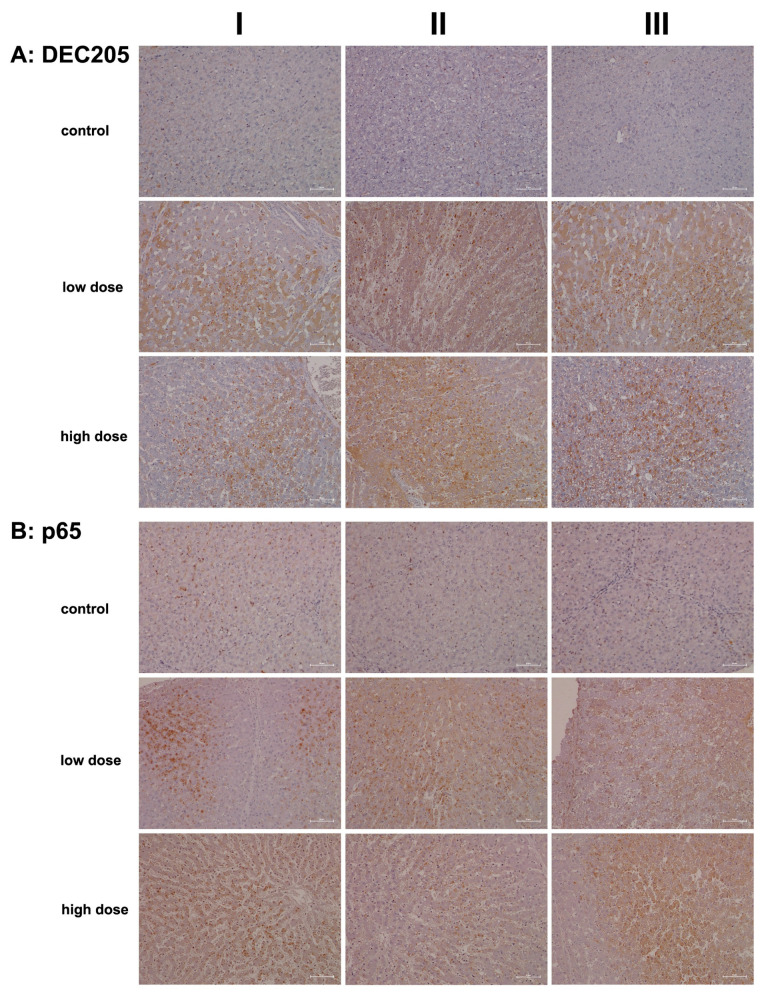
Immunohistochemistry of DEC205 and the RELA protein p65 in liver sections of control and diclofenac-treated animals after daily dosing for 28 days. DEC-205 is a recognition receptor for apoptotic and necrotic cells. Conversely, the transcription factor RELA/p65 is part of the NFkB heterodimeric complex and takes on a protective role in cell survival. Depicted in columns I–III are representative images of individual animals. **Panel** (**A**): **DEC205 immunostaining. Control:** Liver sections of individual controls. None express the DEC-205 protein. **Low dose:** Liver sections of low-dose-treated animals. Diclofenac treatment induced hepatic DEC205 expression in damaged hepatocytes, likely to promote their phagocytic clearance. DEC205-positive dendritic cells and monocytes were observed infiltrating inflamed liver lobules (columns I–III). Note that antigens derived from harmed hepatocytes are presented by DEC205-positive dendritic cells to trigger T-cell responses. **High dose:** Liver sections of high-dose-treated animals. Essentially, the findings are similar to the ones obtained at the low dose and are therefore not dose-related. **Panel** (**B**): **p65 immunostaining. Control:** Liver sections of individual controls. With the exception of a few resident macrophages, none of the controls express the p65 protein. **Low dose:** Liver sections of low-dose-treated animals with nuclear and in part cytosolic expression of p65. **High dose:** Liver sections of high-dose-treated animals. Predominantly, macrophages and neutrophils stain positive (columns I–II) with slight to moderate p65 expression by inflamed hepatocytes (column III). The scale bar represents 50 µm.

Figure 8A presents representative images of liver sections from controls. With the exception of a few macrophages and single neutrophils (column III), none of the liver cells, sinusoidal endothelium, or hepatic stellate cells exhibited positive DEC205 staining. In contrast, low-dose diclofenac treatment (columns I–III) resulted in marked expression of the protein among infiltrating immune cells, including a mixture of monocytes, neutrophils, myeloid dendritic cells, and various subpopulations of Kupffer cells. The images in columns I and III show liver sections with predominant immunostaining in zone 2 and 3 hepatocytes, thus emphasizing the intricate interaction between CD302 and DEC-205 in the context of diclofenac-induced hepatitis. Additionally, we observed DEC205-stained immune cell infiltrates in a highly inflamed liver lobule, while liver cells only faintly expressed the protein (low dose, column II).

**Figure 9:**
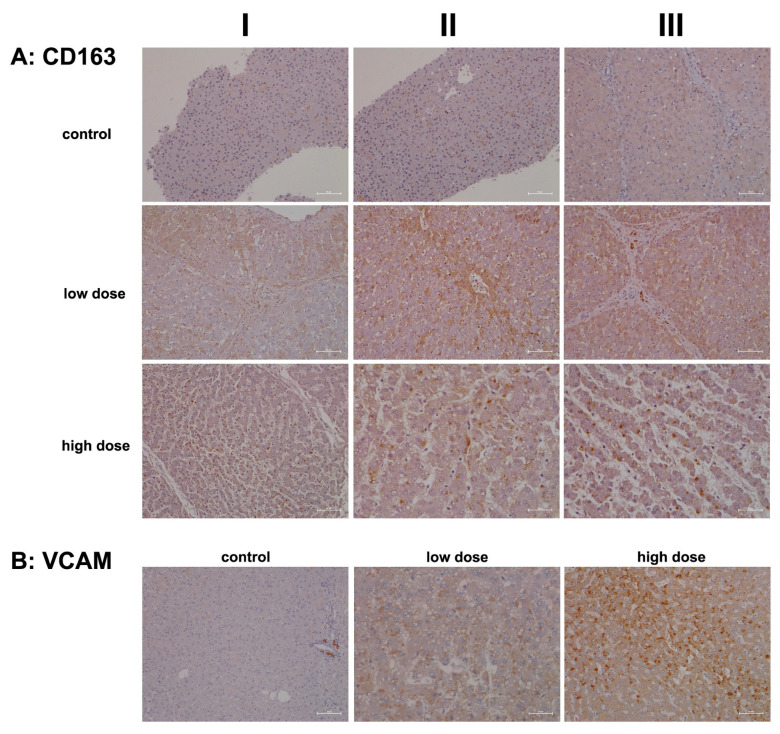
Immunohistochemistry of CD163 and VCAM in liver sections of control and diclofenac-treated animals after daily dosing for 28 days. CD163 is a macrophage and monocyte lineage marker and scavenger receptor that functions in Hb clearance. CD163 supports anti-inflammatory responses. Vascular adhesion molecule 1 (VCAM1) aids the trans-endothelial migration of macrophages during inflammation and facilitates the adhesion of lymphocytes and monocytes at zones of injury. Depicted in columns I–III are representative images of individual animals. **Panel** (**A**): CD163 immunostaining. Control: Liver sections of individual controls. None of the resident macrophages express CD163. **Low dose:** Liver sections of low-dose-treated animals. Diclofenac treatment caused a heterogeneous response among Kupffer cells, and activated macrophages expressed CD163 markedly (column I). Depicted in column III are portal field-infiltrating CD163-positive monocytes. **High dose:** Liver sections of high-dose-treated animals. Depicted are CD163-positive macrophages (columns I–III) but not all macrophages expressed the protein therefore implying a heterogeneous response among resident and activated Kupffer cells. **Panel** (**B**): VCAM immunostaining. Liver sections of a control and a low-, and high-dose-treated animal. Note the VCAM1 positive infiltrates in a portal field surrounding the bile ducts (column I). Diclofenac treatment caused a dose-related increase in VCAM-1 expression of macrophages (low dose, column II) and mixed infiltrates of VCAM1 positive monocytes and lymphocytes in an inflamed hepatic lobule (high dose, column III). The scale bar represents 50 µm, except for **panel** (**A**), high dose, columns II and III (25 µm), and **panel** (**B**), low dose, column II (25 µm).

Importantly, not all hepatocytes expressed CD302, suggesting the presence of subpopulations that likely varied in their expression of DAMPs. DEC-205-positive immune cell infiltrates appear to gather around severely harmed hepatocytes, supporting phagocytosis and cross-presentation to T cells. Evidence indicates that DEC-205 induces T-cell tolerance through the inhibition of CD45 and the upregulation of CTLA-4 [69]. Although we did not observe changes in CD45 or CTLA-4 transcript expression, we noted a significant 2.5-fold upregulation of BCL-6 (Table 1), i.e., a transcription factor that represses CTLA-4 [69]. Additionally, CD27 expression was significantly reduced (Table 1) and CD27 acts as a costimulatory molecule for T cells. We also observed increased CD163 transcript levels (Table 1) and marked protein expression (Figure 9A). This scavenger receptor is upregulated in monocytes and macrophages during inflammation [70].

Collectively, diclofenac elicited a sustained inflammatory response via upregulation of DEC-205 and CD163. However, CD205 expression was not dose-related (high dose, columns I–III). Intriguingly, the monolayer of lipid droplets stained positive as well, and columns I and III show liver lobules with CD205-positive immune cell infiltrates in the absence of marked hepatic CD302 expression. We propose that hepatocytes with high CD302 expression present DAMPs differently, supporting dendritic cell recruitment to injury sites and stimulating adaptive immunity. Indeed, independent research confirmed DEC-205 expression in porcine dendritic cells from various tissues, including the spleen, tonsil, submaxillary, and mesenteric lymph nodes [71]. Hepatocytes that do not express CD302 appear to support an immune response through the lectin complement pathway, with predominantly pro-inflammatory DEC-205-positive Kupffer cells. Together, these findings indicate that diclofenac activates the lectin pathway of the complement system (see also Figure 4 and Figure 5).

Figure 8B shows representative images of liver sections from controls. We occasionally observed faintly positive macrophages and p65-positive neutrophils (columns I–II) distributed throughout the liver lobule. Following low-dose diclofenac treatment (columns I–III), Kupffer cells remained mostly negative. However, diclofenac treatment caused marked expression of p65 in granulocytes/neutrophils (columns I–II), and we observed a few positive monocytes in the marginating pool (columns I–II). p65 acts as a potent transcriptional activator [72], and we identified foci of hepatocytes displaying nuclear p65 staining (EI). We also observed cytosolic p65 expression (low dose, column III, slight to moderate), and shown in column II are p65-positive infiltrating monocytes and Kupffer cells.

A similar staining pattern was seen for the high-dose treatment group, although p65 expression was not dose-related. At the high dose, we observed sinusoidal dilatation and edema (columns I–III), along with mixed infiltrates primarily comprising p65-positive neutrophils, while lymphocytes in the background were mostly negative. This pattern occurred against a backdrop of recurrent regenerative activity in the liver. The image shown in column I illustrates p65-positive immune cell infiltrates. Interestingly, severely harmed hepatocytes showed no p65 expression (columns II–III), and the image shown in column III illustrates a fresh necrotizing lesion in the liver section from a high-dose-treated animal.

In our efforts to characterize the cells involved in diclofenac-induced immune-mediated injury, we investigated the expression of CD163. This marker is prominently associated with monocytes and macrophages and is highly regulated during liver injury [73,74]. We also examined vascular cell adhesion molecule 1 (VCAM-1), which facilitates the trans-endothelial migration of macrophages during inflammation [75]. Importantly, during sterile inflammation, DAMPs enhance CD163 expression, and its shedding can lead to significantly increased plasma concentrations in patients with acute liver failure and acute-on-chronic liver failure [73,74]. The induced expression of CD163 in macrophages is part of an anti-inflammatory response [70].

Figure 9A shows representative images of liver sections from controls, where Kupffer cells exhibited slight positivity for CD163, and endothelial cells displayed minimal expression. Similar findings have been noted in patients with viral liver disease [76]. CD163+ macrophages have been implicated in promoting angiogenesis and vascular permeability, particularly in the context of human atherosclerotic lesions. Additionally, endothelial cells surrounded by CD163+ macrophages show elevated VCAM-1 expression [77].

Following low-dose diclofenac treatment (columns I–III), there was a notable stimulation of CD163 expression in monocytes and Kupffer cells. The liver section shown in column III illustrates monocytic infiltrates in a portal triad with marked expression of the protein. Interestingly, some hepatocytes within inflamed liver lobules also stained positive. This finding is perplexing, given that CD163 is a specific marker for monocytes and macrophages and functions as a scavenger receptor for the hemoglobin–haptoglobin complex. Haptoglobin is secreted by hepatocytes in response to hemolysis, vascular injury, and inflammation. Generally, macrophages internalize the hemoglobin–haptoglobin complex for lysosomal degradation, converting biliverdin to bilirubin. However, hepatocytes can also internalize these complexes via transcytosis [78,79,80,81]. This may explain the positive staining observed in some hepatocytes.

The mosaic-like expression pattern suggests that subpopulations of hepatocytes exhibit significant differences in their uptake of hemoglobin–haptoglobin complexes, with varying staining intensities likely indicating degrees of inflammation. CD163 expression is primarily augmented by anti-inflammatory cytokines, suggesting that CD163+ macrophages may contribute to an attenuation of drug-induced inflammation [82].

High-dose diclofenac treatment resulted in marked increases in CD163+ macrophages and monocytes. These CD163+ macrophages are known to promote the expression of endothelial VCAM-1 [77], facilitating the adhesion of lymphocytes and monocytes to injury sites. We assessed VCAM-1 expression in control and diclofenac-treated animals. Typically, VCAM-1 expression is confined to endothelial cells; however, during inflammation, macrophages and dendritic cells may also express the protein [75]. In the liver section from a vehicle-treated control animal we observed VCAM-1 positive infiltrates in a portal field surrounding the bile duct (Figure 9B). We noted a dose-related increase in VCAM-1 expression in tissue macrophages. As seen in the liver section of a high-dose-treated animal, diclofenac treatment caused substantial mixed infiltrates of VCAM-1+ monocytes and lymphocytes, along with hepatic stellate cells, in an inflamed hepatic lobule.

## 3. Discussion

Drug-induced immune-mediated liver injury is a complex process where drugs and their metabolites acquire immunogenic potential, leading to improper programming of both innate (non-specific) and adaptive (specific) immune cells. Nonsteroidal anti-inflammatory drugs (NSAIDs) are particularly known for hypersensitivity reactions, often manifesting as skin rashes. Diclofenac, in particular, is frequently associated with elevations in aminotransferases, as demonstrated in a prospective clinical trial involving 17,289 patients with arthritis [9]. Moreover, Bjornsson and colleagues identified diclofenac as the second most commonly implicated drug in cases of drug-induced liver injury (DILI) within the general population of Iceland [10].

Diclofenac-induced hypersensitivity reactions are characterized by eosinophilia. Previously, we reported significant increases in peripheral blood eosinophil and neutrophil counts in a canine model of immune-allergic liver injury [5]. In minipigs, we observed similar dose-related increases in neutrophil and monocyte counts [12]. However, the increases in eosinophils and basophils did not reach statistical significance. There is evidence that diclofenac causes eosinophilia in patients, with blood smears and liver biopsies showing higher eosinophil counts in those who recovered from drug-induced liver injury (DILI) [83]. Additionally, NSAIDs, including diclofenac, have been implicated in the DRESS syndrome, i.e., drug rash with eosinophilia and systemic symptoms [84,85].

Based on detailed immunopathology investigations, we demonstrate that diclofenac elicits robust innate immune responses, prominently marked by strong hepatic expression of macrophage migration inhibitory factor (MIF) and its high-affinity receptor, CD74 (Figure 2). CD74, a non-classical MHC class II molecule, is not only crucial for antigen presentation and activation of T- and B-cell responses but also plays a pivotal role in regulating macrophage function during inflammation [26,27]. Beyond antigen presentation, CD74 activates key intracellular signaling cascades, including MAPK/ERK, AMPK, and NF-κB pathways [27]. Our previous transcriptomic analysis in diclofenac-treated minipigs revealed significant upregulation of MAPK6, MAPK14, and MAPKAPK3 [12], thus providing additional mechanistic support for CD74-mediated pathway activation.

We further hypothesize that damaged hepatocytes initiate lipophagy—the lysosomal degradation of intracellular lipid droplets—as a compensatory response to reduce lipid accumulation. MIF, through its interaction with CD74, is known to exert hepatoprotective effects in the context of fatty liver degeneration [25]. Conversely, neutralization of CD74 abolishes MIF’s protective effects, suggesting that the upregulation of CD74 may contribute to hepatocyte survival under lipotoxic stress. These findings align with recent reviews highlighting the roles of MIF and CD74 in tissue protection and wound healing [86]. Supporting their clinical relevance, elevated serum levels of soluble CD74 have been reported in patients with autoimmune hepatitis (AIH) and primary biliary cholangitis (PBC) [87].

In addition to MIF-CD74 signaling, diclofenac activated other innate immune pathways, as evidenced by upregulation of complement system components (Figure 4 and Figure 5), lysozyme (Figure 6), calprotectin (Figure 7), and the macrophage scavenger receptor CD163 (Figure 9). Concurrently, we observed strong regulation of markers of adaptive and humoral immune activation, including significant hepatic expression of immunoglobulins IgG and IgM (Figure 3), upregulation of the acute-phase protein serum amyloid A1 (SAA1) (Figure 7), increased expression of the mannose receptor DEC-205, and marked induction of the transcription factor RelA/p65 (Figure 8), a key effector of NF-κB-mediated immune regulation.

Emerging evidence shows that human hepatocytes are capable of expressing IgG, and siRNA-mediated knockdown of IgG has been shown to suppress cell proliferation and induced apoptosis [28]. This supports our hypothesis that diclofenac-induced hepatic IgG synthesis may play a protective role, potentially by promoting local immune tolerance. Furthermore, a distinct IgG and IgM autoantibody profile has been shown to differentiate drug-induced liver injury (DILI) with autoimmune features from classical autoimmune hepatitis [88]. Elevated IgG levels have also been identified as an independent predictor of liver decompensation and reduced overall survival in patients with nonalcoholic steatohepatitis [89].

Our findings regarding DEC-205 are of particular significance. Beyond its established role in antigen uptake and cross-presentation, the proteolytic shedding of the DEC-205 receptor can provoke an inflammatory response. Evidence suggests that the soluble mannose receptor (sMR), a related molecule, promotes pro-inflammatory macrophage activation. This mechanism involves sMR binding to CD45, leading to an inhibition of CD45 phosphatase activity [90]. Consequently, the Src kinase is activated, triggering the AKT/NF-κB signaling pathway, which further promotes macrophage polarization and pro-inflammatory responses [64].

Additionally, NF-κB is a central transcription factor in immune and inflammatory pathways. It functions as a DNA-binding complex composed of homo- and heterodimers of Rel family proteins, with RelA (p65) serving as a key DNA-binding subunit [91]. The canonical NF-κB pathway plays a pivotal role in hepatitis by driving the transcription of pro-inflammatory genes [92]. During liver injury, a paracrine signaling loop is established in which activated Kupffer cells secrete TNF-α. This cytokine binds to TNF-α receptors on hepatocytes, inducing the dissociation of the p65/β-catenin complex—an event that can influence cell fate, either promoting survival or triggering cell death [93]. Notably, conditional deletion of RelA in hepatocytes sensitizes them to TNFα-induced apoptosis, underscoring the protective role of RelA/p65 in maintaining hepatocyte viability during inflammatory stress [94].

Furthermore, immunogenomics evidenced >eightfold induced β-defensin, nearly sixfold increased lysozymes, and significantly increased expression of certain chemokines to stimulate immune cell migration to zones of hepatic injury. Notwithstanding, the expression of the proinflammatory cytokine CCL8 was repressed as was the expression of CXCL16, which is highly regulated in LPS-stimulated hepatic inflammation (Table 1 and Table 2). Importantly, β-defensins play a key role in preventing neutrophil apoptosis and function as pro-inflammatory mediators in the immune response [95], and both ß-defensin and lysozyme are components of an inflammasome [18].

The fourfold increased expression of the macrophage inflammatory protein-2 (MIP-2)/CXCL2 attracts polymorphonuclear leukocytes (PMLs) to sites of injury [4,96,97]. Activated PMLs release various cytotoxic factors that trigger oxidative stress and cellular damage [98,99,100]. In minipigs, blood smears and histopathology indicated that neutrophils were the predominant cell type in diclofenac-induced liver injury, as reported in our previous study [12], and similar results were observed in rats following diclofenac treatment [97].

There have been case reports of delayed hypersensitivity reactions associated with diclofenac use [101]. One study examined antibody responses to diclofenac and some of its metabolites in a cohort of 59 patients who experienced hypersensitivity reactions [102]. The study found limited evidence for an IgE-mediated response and concluded that prominent metabolites were unlikely to be involved in diclofenac hypersensitivity. Notwithstanding, autoantibodies and drug- or metabolite-dependent antibodies were reported for two patients with alleged diclofenac-mediated immune hemolysis [103].

In the present study, CXCL13 was mildly induced (1.5-fold, *p* < 0.05), potentially stimulating the homing and motility of B cells to sites of injury [104,105]. Conversely, and as described above, expression of CXCL16 was significantly repressed to about 60% of control levels. This chemokine is important for the recruitment of natural killer (NK) T cells and plays a crucial role in the initiation and progression of hepatic inflammation and fibrosis [106]. We interpret its repression as an adaptive response aimed at mitigating damage caused by hepatic inflammation. CXCL16 may play a broader role in drug-induced inflammation, and CXCL16 deficiency in mice reduces acetaminophen-induced hepatotoxicity by decreasing hepatic oxidative stress and inflammation [107]. Moreover, blocking CXCL16 activity with an antibody significantly reduced liver-infiltrating T lymphocytes to zones of injury following LPS treatment of mice [108].

The role of chemokines in liver disease has been reviewed [109]. In the present study, the expression of CCL8/monocyte chemotactic protein (MCP-2) was repressed to 40% of control levels. This cytokine binds to the chemokine receptors CCR1, CCR2B, CCR3, and CCR5 and stimulates the trafficking of monocytes, T cells, NK cells, eosinophils, and basophils to sites of inflammation [110]. Together, MCP-2 is critically important in liver inflammation [111,112,113], and we interpret its downregulation as an effort to mitigate the harmful effects of inflammation. In addition, the interleukin receptor ß subunit IL10RB was upregulated about threefold, while the alpha subunit remained unchanged. Both subunits form a heterotetrameric receptor complex, and upon activation by its ligand IL10, they inhibit pro-inflammatory responses [114]. However, IL10 transcript expression was unchanged, as were most IL10-responsive genes. In fact, the minor but statistically significant upregulation of JAK3 and MYD88 (Table 1) suggests that the IL10/IL10R signaling pathway was inactive.

Activation of complement factors provides compelling evidence for hypersensitivity reactions. Our findings indicate engagement of both the classical and alternative pathways, including a twofold increase in C3 convertase—an essential enzyme complex responsible for cleaving C3 [115]. This was accompanied by elevated levels of anaphylatoxins, which further activated terminal complement components, underscoring the involvement of the complement cascade in diclofenac-induced hypersensitivity. Importantly, diclofenac causes mitochondrial injury, and the resulting mitochondrial damage-associated molecular patterns (mtDAMPs) can activate the complement system. A recent review [116] described a bidirectional interplay between mtDAMPs and complement activation, suggesting a self-perpetuating cycle of mitochondrial dysfunction and immune amplification.

### Study Limitations

First, while minipigs serve as a valuable model for studying human immune-mediated diseases due to numerous physiological and immunological similarities, species-specific differences—particularly in T-cell populations, MHC (SLA) presentation, and innate immune receptor function—must be considered when interpreting results and translating findings to patients. Second, although the 3 mg/kg dose of diclofenac is clinically relevant and reflects realistic patient exposure, we acknowledge that the 15 mg/kg dose represents a supratherapeutic dose. Notwithstanding, it is important to note that all observed immune responses were triggered by the 3 mg/kg dose, and some effects were not even dose-dependent.

## 4. Materials and Methods

### 4.1. Animals

Details regarding the animal study were recently reported [12]. Essentially, this is a trinational project between PWG Genetics Pte Ltd. in Singapore, the Korea Institute of Toxicology (KIT) in Daejeon, Republic of Korea, and Hannover Medical School, Germany.

The animal study was performed by PWG Genetics Pte Ltd. in Singapore, and the laboratory is accredited by the Association for Assessment and Accreditation of Laboratory Animal Care. The study complied with the principles of Good Laboratory Practice and followed the OECD guideline for repeated-dose toxicity study in non-rodent species. Ethical approval was obtained according to Singapore law (protocol approval code: GP11002/2011). Prior to drug treatment, nine specific pathogen-free (SPF) male miniature pigs (Sus scrofa) were adapted to the animal husbandry environment, i.e., a temperature of 20–30 °C, a humidity of 50–80%, air circulation of 15 times/hour, and a 12 h light/dark cycle at 150–300 lux. The animals were fed certified food pellets of 300 g/day (T.S. Corporation, Incheon, Republic of Korea), and water was given ad libitum.

### 4.2. Drug Treatment

We obtained sodium diclofenac (CAS No: 15307-79-6) from Sigma-Aldrich and encapsulated the drug in hard gelatin capsules (Size # 12, Torpac Inc., Fairfield, NJ, USA). The study included three control animals treated with one empty capsule per day as a vehicle control, while the diclofenac treatment group comprised three animals per dose, administered either 3 mg/kg (low dose) or 15 mg/kg (high dose) for 28 days.

Rationale for dose selection: The doses of 3 and 15 mg/kg/day were chosen based on findings from a two-week dose-range-finding (DRF) study, which identified 28 mg/kg/day as the maximum tolerated dose (MTD). Our previous research demonstrated the pathogenesis of diclofenac-induced immunoallergic hepatitis in a canine model of liver injury, and the low dose of 3 mg/kg in minipigs corresponds to the high dose used in the canine study [5]. Additionally, the maximum daily dose for rheumatoid arthritis is approximately 3.3 mg/kg (equivalent to 200 mg diclofenac for a 60 kg individual), making our dose selection relevant to clinical settings.

Throughout the study, we monitored body weight and food consumption, and we euthanized the animals via exsanguination under deep barbiturate anesthesia (Thiopental sodium). Further details can be found in [12].

### 4.3. RNA Extraction

Liver samples from both control and diclofenac-treated animals were surgically excised and snap-frozen in liquid nitrogen. Prior to RNA extraction, the tissues were homogenized using a TissueLyser (Qiagen, Hilden, Germany) following the manufacturer’s guidelines. Total RNA was then isolated using the RNase Mini Kit (Qiagen, Germany) in accordance with the manufacturer’s instructions. The concentration of the total RNA was measured with a NanoDrop spectrophotometer (NanoDrop Technologies, Wilmington, DE, USA), and RNA integrity was assessed using the 2100 Bioanalyzer (Agilent Technologies, Santa Clara, CA, USA).

### 4.4. Microarray Experiments and Data Analysis

We conducted whole genome expression profiling using the Affymetrix porcine GeneChip microarray system (Affymetrix, Santa Clara, CA, USA). All steps, including cDNA synthesis, biotin labeling, fragmentation of cRNA, hybridization, staining, washing, and scanning with a GeneChip Scanner 3000 (Affymetrix, Santa Clara, CA, USA), were performed as previously described [4,5,117].

Data normalization was carried out using the MAS5 algorithm, and differentially expressed genes (DEGs) were identified through hypergeometric tests with the criteria of fold change > 1.5 and *p*-value < 0.05. We controlled the false discovery rate (FDR) at α = 0.05, considering only *p*-values corrected by the Benjamini–Hochberg procedure. Additional details can be found in part I of our diclofenac study [12].

### 4.5. Immunohistochemistry

Livers from both control and diclofenac-treated animals were fixed in 4% buffered paraformaldehyde and embedded in paraffin blocks using standard laboratory protocols. Sections measuring 1–2 μm in thickness were deparaffinized and rehydrated through a descending alcohol series, followed by a 4 min wash in distilled H_2_O. Antigen retrieval was then performed in citrate buffer (pH 6) or Tris-EDTA buffer (pH 9.0) in a water bath at 98 °C for 30 min.

For immunohistochemistry, we utilized the ZytoChem-Plus HRP Polymer Kit from Zytomed Systems (Berlin, Germany). The slides were rinsed with distilled H_2_O, followed by a 5-minute incubation in tris-buffered saline (washing buffer). Endogenous peroxidase activity was blocked using a 3% peroxidase blocking reagent (Merck, Darmstadt, Germany) for 5 min, followed by a second wash. We then applied a protein-block serum-free reagent for 5 min (ZytoChem-Plus HRP Polymer Kit, reagent 1) and incubated the sections with primary antibodies for 60 min. The antibodies were sourced from various vendors and diluted with washing buffer, as detailed in the table below:
**Antibody****Vendor****Cat No.****Lot Number****Dilution****Antigen** **Retrieval**C1INHSanta Cruzsc-46298B27061:200ph6C3abcamab112829GR119618-21:1000ph6CD163abcamab87099GR3197855-11:100ph6CD74LSanta Cruzsc-5441I28071:500ph6DEC-205Santa Cruzsc-14602L18131:250ph6Factor BSanta Cruzsc-67141G18081:25ph9Factor Habcamab170036GR179267-141:300ph6IgGDakoA0423200200591:10,000PronaseIgMDakoA0425865311:1000PronaseKLF6Santa Cruzsc-7158D26131:50ph6Lysozymeabcamab74666GR3185036-1RTUph6MIFAbbiotec251415130304011:500ph9NF-kB p65abcamab86299GR3204852-31:1500ph9S100A9abcamab63818GR126635-11:300ph6SAA1abcamab171030GR147621-101:100ph6VCAM-1Santa Cruzsc-1504H12151:50ph6

We incubated the bound primary or bridging antibody with the labeled polymer HRP anti-rabbit or anti-mouse secondary antibody (ZytoChem-Plus HRP Polymer Kit, reagent 2) for 20 min. Following this, we added reagent 3 from the ZytoChem-Plus HRP Polymer Kit and placed the slides in a moist chamber at room temperature for an additional 30 min.

After completing the HRP reaction, the sections were counterstained with Hematoxylin for 5 min, followed by washing the slides under running warm tap water for 10 min. The sections were then dehydrated in a cabinet at 60 °C for 20 min. Finally, the slides were coverslipped and examined under a light microscope (Nikon Ni-E microscope, Tokyo, Japan), with images captured using Nikon NIS Basic Research Microscopic Imaging Software version 4.3.

## 5. Conclusions

Our findings demonstrate that diclofenac triggers robust innate immune responses, evidenced by marked expression of macrophage migration inhibitory factor, CD74, components of the complement system, lysozyme, calprotectin, and CD163. Furthermore, we provide compelling evidence that diclofenac elicits adaptive and humoral immune activation, highlighted by significant hepatic expression of IgG and IgM, induction of the acute phase protein serum amyloid A1, upregulation of the mannose receptor DEC-205, and increased expression of the transcription factor RelA/p65—a central regulator of immune responses. Collectively, these results underscore diclofenac’s capacity to provoke immune-mediated liver injury at clinically relevant doses.

From a clinical perspective, while dose reduction may modestly reduce the risk, the most prudent approach is to avoid diclofenac in patients with a known or suspected hypersensitivity risk. If its use is deemed necessary, it should be administered at the lowest effective dose, with appropriate monitoring and informed consent from patients.

## Figures and Tables

**Table 1:** Significantly regulated genes coding for immune and inflammatory response and leukocyte migration. Minipigs were given 15 of mg/kg diclofenac daily for 28 days. We performed whole genome hepatic transcript expression profiling, and DEGs were calculated based on the criteria fold change > 1.5 and an FDR-adjusted *p*-value < 0.05.

Immune/Inflammatory Response/Leukocyte Migration		
Probeset ID	Gene Symbol	Fold Change X ± SD
Ssc.27693.1.A1_a_at	ADCY8	−1.51 ± 0.08
Ssc.6943.1.A1_at	ANGPT1	−1.51 ± 0.39
Ssc.3703.1.S1_at	APOA2	−1.57 ± 0.07
Ssc.14503.1.S1_at	APOA4	1.56 ± 0.29
Ssc.8659.2.S1_a_at	ATG12	1.53 ± 0.15
Ssc.17615.1.S1_at	ATP1B1	1.5 ± 0.37
Ssc.12348.2.S1_at	B2M	−1.53 ± 0.34
Ssc.15518.1.A1_at	BCL6	2.38 ± 0.48
Ssc.8594.1.A1_at	BLNK	−1.74 ± 0.48
Ssc.9957.1.A1_at	CCL8	−2.43 ± 1.23
Ssc.5053.1.S1_at	CD163	2.04 ± 0.42
Ssc.19817.1.S1_at	CD27	−1.53 ± 0.15
Ssc.26104.1.S1_at	CD46	1.63 ± 0.35
Ssc.3593.1.S1_at	CTSH	−1.72 ± 0.53
Ssc.22002.2.A1_at	CXCL13	1.54 ± 0.16
Ssc.19692.1.S1_at	CXCL2	2.56 ± 1.05
Ssc.15885.1.S1_at	DDX58	−1.57 ± 0.07
Ssc.21145.1.S1_at	DEFB1 (PBD2)	8.32 ± 4.48
Ssc.2714.3.S1_at	FYN	−1.56 ± 0.24
Ssc.6646.1.S1_at	GRB7	−1.54 ± 0.19
Ssc.376.1.S1_at	HAMP	−1.8 ± 0.25
Ssc.7558.1.A1_at	HERC6	−1.69 ± 0.23
Ssc.12191.1.A1_at	HSP90AA1	2.01 ± 0.3
Ssc.10588.1.A1_at	IFI44L	−2.03 ± 0.41
Ssc.5955.1.A1_at	IL10RB	2.6 ± 0.28
Ssc.528.1.S1_at	IL5	−1.52 ± 0.15
Ssc.286.1.S1_s_at	IRG6 (RSAD2)	−3.24 ± 1.23
Ssc.11557.1.A1_at	ISG15	−1.86 ± 0.11
Ssc.23054.1.S1_at	JAK3	1.52 ± 0.19
Ssc.18557.1.S1_at	KNG1	−1.61 ± 0.15
Ssc.15980.1.S1_at	LBP	1.87 ± 0.76
Ssc.14340.1.S1_at	LITAF	1.81 ± 0.22
Ssc.670.1.S1_at	LYZ	5.89 ± 1.04
Ssc.18928.1.A1_at	MADCAM1	1.71 ± 0.38
Ssc.13711.1.S1_at	MAP2K6	−1.61 ± 0.13
Ssc.24291.1.A1_s_at	MAPK14	1.53 ± 0.08
Ssc.3033.1.S1_a_at	MAPKAPK3	1.55 ± 0.11
Ssc.18868.1.S1_at	MBL1 (MBL2)	−2.61 ± 1.59
Ssc.6463.2.S1_at	MYD88	1.53 ± 0.19
Ssc.1031.1.S1_at	OAS1	−2.04 ± 0.66
Ssc.10256.1.A1_at	PDE4B	1.59 ± 0.07
Ssc.16110.1.A1_at	PIAP (BIRC3)	1.64 ± 0.23
Ssc.11206.1.A1_at	PLCG1	−1.54 ± 0.07
Ssc.9170.1.A1_at	PRKD1	−2.52 ± 0.42
Ssc.23963.1.S1_at	RGC32 (RGCC)	−2.87 ± 1.39
Ssc.2381.1.A1_at	S100A9	4.13 ± 2.55 *
Ssc.18849.1.A1_at	SH2D1A	−1.52 ± 0.09
Ssc.222.1.S1_at	SLA-DRA (HLA-DRA)	−1.54 ± 0.26
Ssc.6583.1.S1_at	SR-PSOX (CXCL16)	−1.51 ± 0.15
Ssc.2594.1.S1_at	SUGT1	1.56 ± 0.11
Ssc.16640.1.A1_at	VSIG4	1.58 ± 0.17
Ssc.27161.1.A1_at	ZC3HAV1	−1.57 ± 0.13

* *p* < 0.05.

**Table 2:** Significantly regulated genes coding for glucocorticoid and cytokine-mediated signaling pathways and interferon-γ signaling. Minipigs were given 15 mg/kg daily for 28 days. We performed whole genome hepatic transcript expression profiling, and DEGs were calculated based on the criteria fold change > 1.5 and an FDR-adjusted *p*-value < 0.05.

Response to Glucocorticoid Stimulus		
Probeset ID	Gene Symbol	Fold Change (Average) ± SD
Ssc.3703.1.S1_at	APOA2	−1.57 ± 0.07
Ssc.5737.1.S1_at	CDKN1A (p21)	2.74 ± 1.11
Ssc.14393.2.S1_x_at	HSD3B1	−1.91 ± 0.14
Ssc.16231.3.S1_a_at	IGF1	1.52 ± 0.19
Ssc.47.1.S1_at	IGFBP2	−1.85 ± 0.37
Ssc.15986.2.A1_at	INSR	−1.55 ± 0.07
Ssc.7297.1.S1_at	MAOB	−1.86 ± 0.22
Ssc.6988.1.A1_at	PAM	−1.51 ± 0.29
Ssc.9781.1.S1_at	SERPINE1	1.58 ± 0.04
Ssc.2464.1.S1_at	STC1	1.58 ± 0.35
Ssc.14066.2.S1_at	TAT	−2.15 ± 0.45
Ssc.21161.1.S1_at	UGT1A6	−2.17 ± 0.44
**Cytokine-Mediated Signaling Pathway**		
**Probeset ID**	**Gene Symbol**	**Fold Change (Average) ± SD**
Ssc.12348.2.S1_at	B2M	−1.53 ± 0.34
Ssc.28997.1.S1_at	CSF2RB	1.51 ± 0.11
Ssc.29054.1.A1_at	GBP1	−2.55 ± 0.54
Ssc.9565.1.S1_at	IFNGR1	1.55 ± 0.18
Ssc.528.1.S1_at	IL5	−1.52 ± 0.15
Ssc.12504.1.A1_at	ISG12 (IFI27)	−2.45 ± 0.82
Ssc.11557.1.A1_at	ISG15	−1.86 ± 0.11
Ssc.23054.1.S1_at	JAK3	1.52 ± 0.19
Ssc.5991.1.A1_at	KRT18	1.67 ± 0.25
Ssc.263.1.S1_at	LEPR	1.59 ± 0.27
Ssc.15640.1.S1_at	MT2A	1.65 ± 0.37
Ssc.6463.2.S1_at	MYD88	1.53 ± 0.19
Ssc.1031.1.S1_at	OAS1	−2.04 ± 0.66
Ssc.11206.1.A1_at	PLCG1	−1.54 ± 0.07
Ssc.222.1.S1_at	SLA-DRA (HLA-DRA)	−1.54 ± 0.26
Ssc.7207.3.A1_at	SP100	1.74 ± 0.57
Ssc.336.1.S1_at	USP18	−2.25 ± 0.11
**Response to Interferon-Gamma**		
**Probeset ID**	**Gene Symbol**	**Fold Change (Average) ± SD**
Ssc.12348.2.S1_at	B2M	−1.53 ± 0.34
Ssc.6583.1.S1_at	SR-PSOX (CXCL16)	−1.51 ± 0.15
Ssc.7362.1.S1_at	EPRS	1.61 ± 0.33
Ssc.29054.1.A1_at	GBP1	−2.55 ± 0.54
Ssc.222.1.S1_at	SLA-DRA (HLA-DRA)	−1.54 ± 0.26
Ssc.9565.1.S1_at	IFNGR1	1.55 ± 0.18
Ssc.15640.1.S1_at	MT2A	1.65 ± 0.37
Ssc.1031.1.S1_at	OAS1	−2.04 ± 0.66
Ssc.23553.1.S1_at	SEC61A1	1.58 ± 0.24
Ssc.7207.3.A1_at	SP100	1.74 ± 0.57
**Interferon-Gamma-Mediated Signaling Pathway**		
**Probeset ID**	**Gene Symbol**	**Fold Change (Average) ± SD**
Ssc.12348.2.S1_at	B2M	−1.53 ± 0.34
Ssc.29054.1.A1_at	GBP1	−2.55 ± 0.54
Ssc.222.1.S1_at	SLA-DRA (HLA-DRA)	−1.54 ± 0.26
Ssc.9565.1.S1_at	IFNGR1	1.55 ± 0.18
Ssc.15640.1.S1_at	MT2A	1.65 ± 0.37
Ssc.1031.1.S1_at	OAS1	−2.04 ± 0.66
Ssc.7207.3.A1_at	SP100	1.74 ± 0.57

**Table 3:** Significantly regulated genes coding for cell death signaling. Minipigs were given 15 mg/kg daily for 28 days. We performed whole genome hepatic transcript expression profiling, and DEGs were calculated based on the criteria fold change > 1.5 and an FDR-adjusted *p*-value < 0.05.

Cell Death		
Probeset ID	Gene Symbol	Fold Change X ± SD
Ssc.6943.1.A1_at	ANGPT1	−1.51 ± 0.39
Ssc.8980.1.A1_at	ANGPTL4	−1.54 ± 0.29
Ssc.14212.1.A1_at	ANKRD13C	−1.65 ± 0.17
Ssc.15518.1.A1_at	BCL6	2.38 ± 0.48
Ssc.16110.1.A1_at	BIRC3	1.64 ± 0.23
Ssc.6833.1.S1_at	BTG1	−1.52 ± 0.08
Ssc.717.1.S1_at	CCK	−1.52 ± 0.03
Ssc.19817.1.S1_at	CD27	−1.53 ± 0.15
Ssc.5737.1.S1_at	CDKN1A (p21)	2.74 ± 1.11
Ssc.6966.3.S1_a_at	CDKN1B (p27)	−2.39 ± 0.96
Ssc.21845.2.S1_at	C-FLIP (CFLAR)	1.59 ± 0.29
Ssc.3593.1.S1_at	CTSH	−1.72 ± 0.53
Ssc.22002.2.A1_at	CXCL13	1.54 ± 0.16
Ssc.11184.1.S1_at	DAD1	1.56 ± 0.33
Ssc.22064.1.S1_at	DFFA	−1.55 ± 0.17
Ssc.10498.1.A1_at	EAF2	2.56 ± 0.21
Ssc.4303.1.S1_at	EEF1E1	1.55 ± 0.21
Ssc.2714.3.S1_at	FYN	−1.56 ± 0.24
Ssc.31027.1.A1_at	G2E3	1.52 ± 0.16
Ssc.17033.1.S1_at	HIGD2A	−1.53 ± 0.18
Ssc.2667.1.S1_a_at	HRG	−2.26 ± 0.53
Ssc.1241.1.S1_at	HSPE1	1.58 ± 0.15
Ssc.1231.1.A1_at	HSPH1	1.77 ± 0.48
Ssc.16231.3.S1_a_at	IGF1	1.52 ± 0.19
Ssc.15588.1.S2_at	IGFBP3	−1.65 ± 0.13
Ssc.12504.1.A1_at	IFI27	−2.45 ± 0.82
Ssc.23054.1.S1_at	JAK3	1.52 ± 0.19
Ssc.27622.1.S1_at	KLF11	−1.88 ± 0.38
Ssc.18557.1.S1_at	KNG1	−1.61 ± 0.15
Ssc.5991.1.A1_at	KRT18	1.67 ± 0.25
Ssc.9655.1.A1_at	LOC100518125 (YBX3)	1.59 ± 0.3
Ssc.13711.1.S1_at	MAP2K6	−1.61 ± 0.13
Ssc.6463.2.S1_at	MYD88	1.53 ± 0.19
Ssc.19546.1.S1_at	NME1	1.53 ± 0.2
Ssc.16864.1.S1_at	PPARGC1A	−2.11 ± 0.46
Ssc.25206.1.S1_at	PPIF	1.74 ± 0.33
Ssc.6371.1.A1_at	PRNP	1.54 ± 0.15
Ssc.12758.1.A1_at	PSMB9	−1.54 ± 0.28
Ssc.830.1.S1_at	PSME2	−1.51 ± 0.26
Ssc.23963.1.S1_at	RGCC	−2.87 ± 1.39
Ssc.9781.1.S1_at	SERPINE1	1.58 ± 0.04
Ssc.7604.1.A1_at	SKIL	1.6 ± 0.41
Ssc.3706.1.S2_at	SOD2	1.91 ± 0.53
Ssc.5930.1.S1_at	SORT1	−1.82 ± 0.76
Ssc.7207.3.A1_at	SP100	1.74 ± 0.57
Ssc.30934.1.S1_at	TERF1	−1.74 ± 0.64
Ssc.6634.1.A1_at	THY1	−1.54 ± 0.38
Ssc.14506.1.S1_at	TOP2A	−1.77 ± 0.33
Ssc.2095.1.S1_at	VEGFB	−1.57 ± 0.09
Ssc.2884.1.S1_at	VIL1	−1.79 ± 0.58

## Data Availability

The data presented in this study are available within the article or its Supplementary Materials.

## Supplementary Materials

The following supporting information can be downloaded at: https://www.mdpi.com/article/10.3390/ijms26125899/s1.

## Abbreviations

ADR	adverse drug reactions
AIH	autoimmune hepatitis
CLR	C-type lectin receptors.
CXCL	C-X-C motif chemokine ligand
DAMP	death-associated molecular pattern
DEGs	differentially expressed genes
DILI	drug-induced liver injury
GR	glucocorticoid receptor
HSC	hepatic stellate cells
IgG	immunoglobulin G
IgE	immunoglobulin E
IgM	immunoglobulin M
MCP-2	monocyte chemotactic protein-2
MIF	macrophage migration inhibitory factor
NSAID	nonsteroidal anti-inflammatory diclofenac
PBC	primary biliary cholangitis
PRRs	pattern recognition receptors
Rgcc	regulator of cell cycle
SAA1	serum amyloid A1
sMR	soluble mannose receptor
TFBS	transcription factor binding sites
VCAM1	vascular cell adhesion molecule 1
