# Supplementary material for: Diclofenac Immune-Mediated Hepatitis: Identification of Innate and Adaptive Immune Responses at Clinically Relevant Doses
Source: Int J Mol Sci. 2025 Jun 19;26(12):5899. doi: 10.3390/ijms26125899 (PMC12192901; doi:10.3390/ijms26125899)
Supplement: Supplementary file 1 [file ijms-26-05899-s001.zip › ijms-3556605-supplementary.pdf]

Supplementary Figure S1: Gene ontology and pathway mapping network of hepatic DEGs in response to low dose diclofenac treatment.

A visualization of hepatic enriched pathway terms and biological processes was computed with the ClueGO and the GeneXplain software of low dose treated animals.

Panel A: Network constructed by the GO plug-in ClueGO of the Cytoscape software.

Panel B: The enriched biological processes and pathways annotated with the GeneXplain platform and visualized using the Cytoscape software version 3.4. The red and green colored nodes illustrate up- and down regulated genes, respectively.

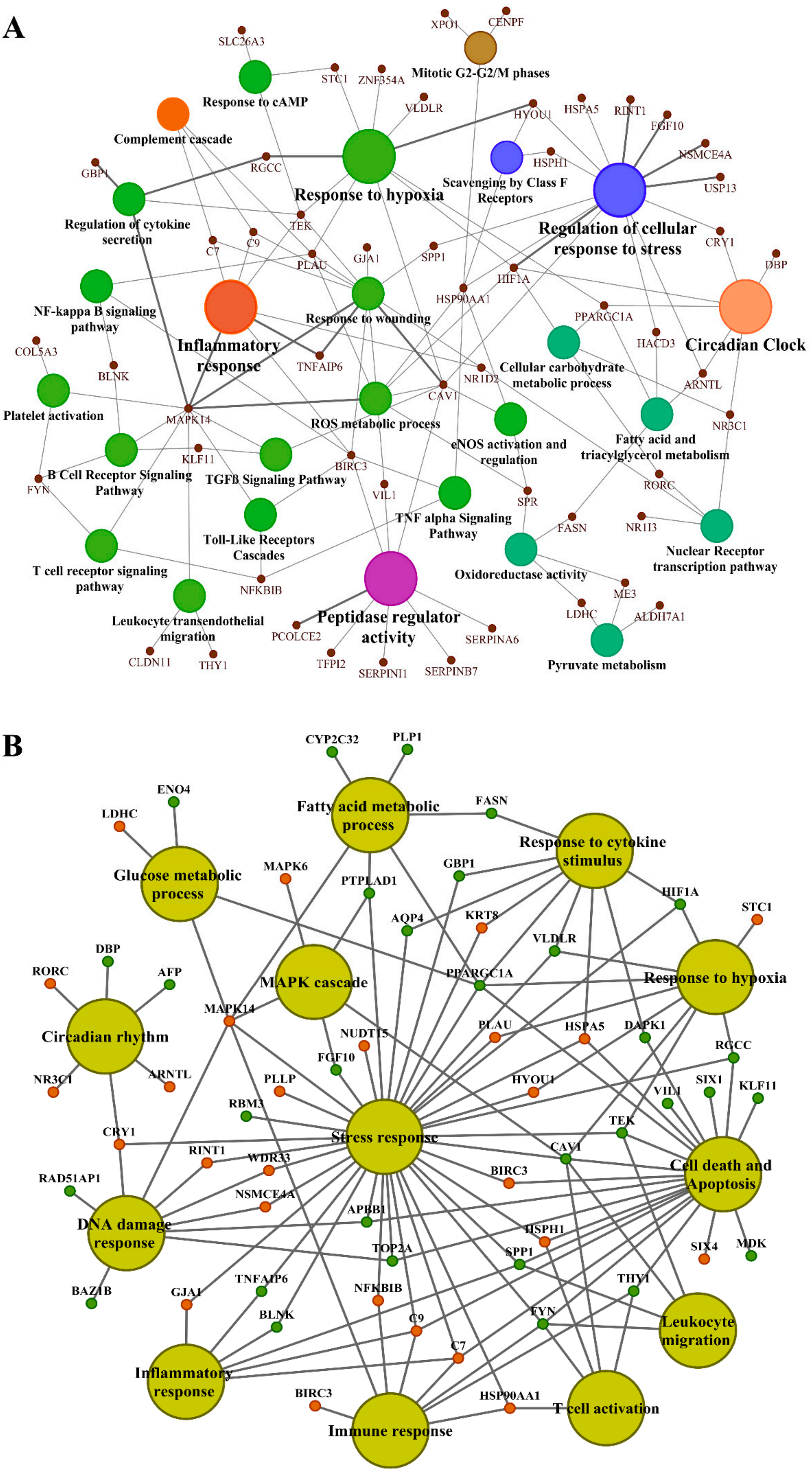
A
XPO1
CENPF
SLC26A3
Mitotic G2-G2/M phases
STC1
ZNF354A
Response to cAMP
VLDLR
HYOU1
HSPA5
RINT1
FGF10
Complement cascade
NSMCE4A
HSPH1
USP13
GBP1
RGCC
Scavenging by Class F Receptors
Response to hypoxia
Regulation of cytokine secretion
TEK
Regulation of cellular response to stress
CRY1
C7
C9
GJA1
SPP1
DBP
PLAU
HIF1A
HSP90AA1
NF-kappa B signaling pathway
Response to wounding
PPARGC1A
Inflammatory response
HACD3
Circadian Clock
COL5A3
TNFAIP6
Cellular carbohydrate metabolic process
NR1D2
BLNK
ARNTL
CAV1
Platelet activation
MAPK14
ROS metabolic process
NR3C1
eNOS activation and regulation
Fatty acid and triacylglycerol metabolism
KLF11
BIRC3
B Cell Receptor Signaling Pathway
TGFB Signaling Pathway
RORC
FYN
VIL1
SPR
TNF alpha Signaling Pathway
Toll-Like Receptors Cascades
FASN
NR1I3
Nuclear Receptor transcription pathway
T cell receptor signaling pathway
Oxidoreductase activity
NFKBIB
ME3
Leukocyte transendothelial migration
Peptidase regulator activity
LDHC
ALDH7A1
PCOLCE2
SERPINA6
CLDN11
THY1
TFPI2
SERPINI1
SERPINB7
Pyruvate metabolism
B
CYP2C32
PLP1
ENO4
LDHC
Fatty acid metabolic process
FASN
Glucose metabolic process
MAPK6
Response to cytokine stimulus
PTPLAD1
GBP1
HIF1A
STC1
AQP4
KRT8
VLDLR
DBP
RORC
AFP
MAPK cascade
PPARGC1A
Response to hypoxia
MAPK14
Circadian rhythm
NUDT15
PLAU
HSPA5
DAPK1
RGCC
FGF10
NR3C1
ARNTL
PLLP
HYOU1
SIX1
KLF11
VIL1
RBM3
TEK
CRY1
Stress response
RINT1
WDR33
CAV1
Cell death and Apoptosis
BIRC3
RAD51AP1
NSMCE4A
APBB1
DNA damage response
HSPH1
TOP2A
SPP1
SIX4
MDK
BAZ1B
TNFAIP6
THY1
GJA1
NFKBIB
BLNK
FYN
Leukocyte migration
C9
C7
Inflammatory response
BIRC3
HSP90AA1
T-cell activation
Immune response
